# Comparative phylogenomic and long-read genomic characterization of an Egyptian ST6-MRSA-IVa clinical isolate within a globally conserved multidrug-resistant lineage

**DOI:** 10.3389/fmicb.2026.1855574

**Published:** 2026-06-08

**Authors:** Mohamed G. Seadawy, Ahmed A. Sanad, Haneen M. Helmy, Dalia Mahmoud El-sherbiny, Hanna Mohamed Elghaiaty, Haidy Hany Elsamman, Farah Tarek Elfayomy, Ahmed Aymen Elhady, Amin M. Eissa, Adel A. El-Morsi

**Affiliations:** 1 Biodefense Center for Infectious and Emerging Diseases, Egyptian Armed Forces, Cairo, Egypt; 2 Biotechnology Department, Faculty of Science, Mansoura University, Mansoura, Egypt

**Keywords:** antimicrobial resistance, long-read sequencing, methicillin-resistant *Staphylococcus aureus* (MRSA), mobile genetic elements, multidrug resistance, phylogenomic analysis, resistome, ST6 lineage

## Abstract

Methicillin-resistant *Staphylococcus aureus* (MRSA) remains a major global public health concern due to its multidrug resistance, extensive genome plasticity, and rapid evolutionary adaptability. In this study, 50 clinical MRSA isolates were screened using antimicrobial susceptibility testing and the VITEK® 2 system to identify multidrug-resistant phenotypes. One isolate (SAMN57098905; MRSA21-2025) exhibiting the broadest multidrug-resistant profile among the analyzed isolates was selected for integrated long-read genomic and comparative phylogenomic characterization. Whole-genome sequencing was performed using Oxford Nanopore Technologies followed by Medaka polishing, genome annotation, antimicrobial resistance profiling, virulence characterization, SCCmec typing, insertion-sequence analysis, prophage identification, multilocus sequence typing (MLST), and comparative phylogenomics against 50 publicly available ST6 genomes. Core-genome maximum-likelihood phylogeny together with Panaroo-based pan-genome reconstruction was applied to investigate evolutionary relatedness and genomic diversification. The near-complete genome assembly (~2.85 Mb; 33.14% GC content) was reconstructed into three contigs with high sequencing depth and assigned to ST6, spa type t304, and SCCmec type IVa (2B). Resistome analysis revealed a predominantly chromosomally encoded multidrug-resistant architecture centered on *mecA* and SCCmec-associated determinants together with multiple efflux-associated and regulatory-associated resistance loci including *norA*, *norC, sdrM, mgrA, arlS*, and *mepA*. Comparative phylogenomic analyses demonstrated that the Egyptian isolate clustered within a geographically distributed ST6-IVa lineage closely related to European and Asian clinical strains, supporting phylogenetic conservation within this clonal background. Pan-genome analysis identified 2,495 core genes within a total pan-genome of 2,680 genes, indicating substantial lineage conservation with accessory genome variability. Mobilome analysis identified 19 insertion-sequence elements distributed across eight IS families, highlighting extensive genome plasticity and potential genome remodeling activity. Two chromosomally integrated prophages were additionally detected, including a Sa3int-like immune evasion prophage carrying the IEC-associated genes *sak* and scn integrated within the *β*-hemolysin locus. Virulence profiling revealed a broad toxin-associated repertoire including *sea*, *hlgABC*, *lukDE*, splA/B/E, and aur, whereas canonical Panton–Valentine leukocidin genes were not fully detected. Collectively, this study provides a high-resolution long-read comparative genomic framework linking multidrug resistance, mobilome composition, prophage-associated virulence, and phylogenetic structure in an Egyptian ST6-MRSA-IVa isolate, highlighting the value of integrated comparative genomics for genomic surveillance and evolutionary tracking of clinically relevant MRSA lineages.

## Introduction

Methicillin-resistant *Staphylococcus aureus* (MRSA) remains one of the most clinically significant bacterial pathogens worldwide due to its extensive antimicrobial resistance, remarkable genomic adaptability, and ability to cause a broad spectrum of infections ranging from skin and soft tissue infections to severe invasive diseases including pneumonia, bacteremia, and infective endocarditis (Chambers and DeLeo, 2009; Turner et al., 2019; Lee et al., 2018). The continued emergence and dissemination of multidrug-resistant MRSA lineages have substantially limited therapeutic options and increased healthcare-associated morbidity, mortality, and economic burden globally. Consequently, improved genomic surveillance and high-resolution molecular characterization strategies are increasingly required to understand the evolutionary dynamics and dissemination of clinically relevant MRSA clones.

The defining feature of MRSA is resistance to *β*-lactam antibiotics mediated primarily through acquisition of the *mecA* gene, which encodes an alternative penicillin-binding protein with reduced affinity for *β*-lactams. However, antimicrobial resistance in MRSA is frequently multifactorial and may additionally involve efflux systems, transcriptional regulators, target modification mechanisms, and accessory resistance determinants that collectively contribute to multidrug-resistant phenotypes (Peacock and Paterson, 2015; Blair et al., 2015). The accumulation of these determinants reflects the considerable adaptive capacity of MRSA under sustained antimicrobial selection pressure.

Phenotypic antimicrobial susceptibility testing remains an essential first-line approach for identifying highly resistant clinical isolates and guiding infection control strategies (Cheesbrough, 2006; Clinical and Laboratory Standards Institute (CLSI), 2024). Automated systems such as the VITEK® 2 compact platform enable rapid resistance profiling and facilitate prioritization of isolates for downstream genomic investigation. Integrating phenotypic resistance data with whole-genome sequencing (WGS) approaches provides a more comprehensive understanding of the genomic determinants underlying antimicrobial resistance, virulence, and bacterial adaptation.

Mobile genetic elements (MGEs) are central drivers of MRSA genome evolution and diversification. Elements including SCCmec cassettes, insertion sequences, transposons, prophages, and genomic islands facilitate horizontal gene transfer and genomic rearrangements that contribute to the dissemination of antimicrobial resistance and virulence-associated determinants (Malachowa and DeLeo, 2010; Partridge et al., 2018). These mobile elements substantially influence genome plasticity and may contribute to the persistence and adaptation of MRSA lineages in both healthcare-associated and community settings.

Recent advances in long-read whole-genome sequencing technologies have significantly improved the resolution of bacterial genomic investigations by enabling near-complete genome reconstruction and more accurate characterization of structurally complex genomic regions such as SCCmec elements, insertion-sequence-associated loci, and prophage regions (Didelot et al., 2012; Koren and Phillippy, 2015). When combined with comparative genomics and phylogenomic analyses, long-read sequencing provides a powerful framework for investigating resistomes, virulomes, mobilomes, and evolutionary relationships among globally distributed MRSA lineages (Wick et al., 2019; Kolmogorov et al., 2019).

Multilocus sequence typing (MLST), spa typing, and comparative phylogenomics are widely used to investigate the population structure, dissemination, and evolutionary relationships of MRSA clones (Gurevich et al., 2013). Certain MRSA lineages have been associated with enhanced antimicrobial resistance, successful clinical dissemination, and lineage-specific virulence characteristics, highlighting the importance of integrating genomic typing with comparative genomic context.

In the present study, 50 clinical isolates were screened using phenotypic antimicrobial susceptibility testing to identify highly resistant strains. One isolate exhibiting an extensive multidrug-resistant phenotype was selected for comprehensive long-read genomic characterization. Integrated analyses including genome assembly, resistome profiling, SCCmec characterization, virulence-associated gene identification, insertion-sequence analysis, prophage detection, pan-genome analysis, and comparative phylogenomics against publicly available ST6 genomes were subsequently performed. This integrated approach provides a high-resolution genomic framework linking phenotypic multidrug resistance with mobilome composition, virulence-associated architecture, and phylogenetic context within an Egyptian ST6-MRSA-IVa lineage.

## Materials and methods

### Overview of the study workflow

#### Ethics approval and consent to participate

**Figure 1:**
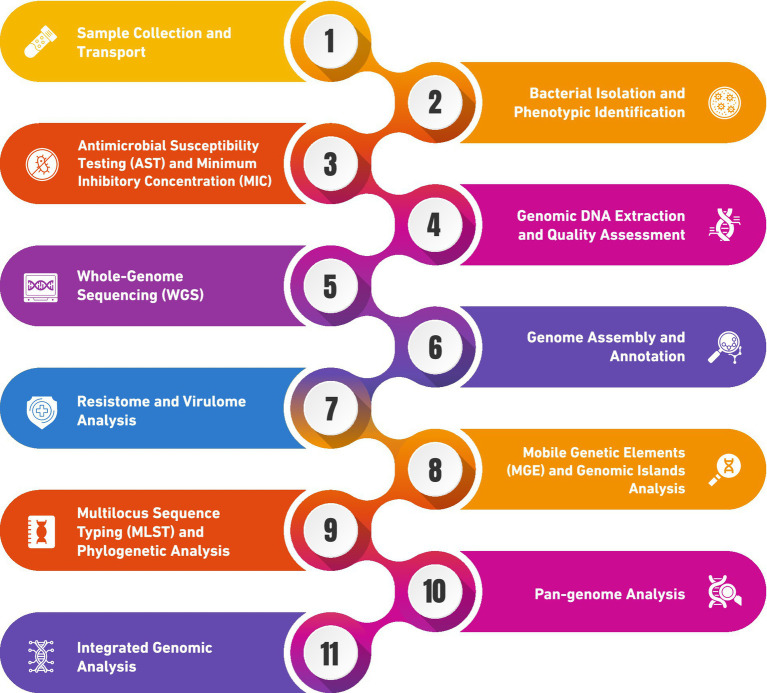
Overview of the study workflow. This figure illustrates the overall workflow of the study, beginning with clinical sample collection, bacterial isolation, and phenotypic antimicrobial susceptibility testing of 50 clinical *Staphylococcus aureus iso*lates. The isolate demonstrating the highest multidrug-resistant phenotype (MRSA21-2025) was selected for long-read whole-genome sequencing using Oxford Nanopore Technologies. Downstream analyses included genome assembly, annotation, resistome profiling, virulence-associated gene characterization, SCCmec typing, insertion-sequence analysis, prophage detection, comparative phylogenomics, and pan-genome reconstruction. All datasets were subsequently integrated to provide comprehensive genomic characterization of the Egyptian ST6-MRSA-IVa isolate.

The study was approved by the Institutional Review Board (IRB) of the Military Medical Academy, Health and Preventive Medicine Institute, Ministry of Defense, Egypt (Approval No. 17–2025). All procedures involving human-derived samples were conducted in accordance with the ethical standards of the institutional research committee and the Declaration of Helsinki. Written informed consent was obtained from all participants prior to sample collection Figure 1.

#### Isolation and identification of the organism

Clinical endotracheal aspirate (ETA) specimens were cultured on blood agar, MacConkey agar, and nutrient agar media (Oxoid, UK) and incubated aerobically at 35 ± 2 °C for 18–24 h (Cheesbrough, 2006). Presumptive *Staphylococcus aureus* isolates were initially identified using conventional microbiological procedures including colony morphology, Gram staining, catalase testing, and coagulase testing.

Species-level confirmation and antimicrobial susceptibility profiling were subsequently performed using the VITEK® 2 compact system (bioMérieux, France) according to the manufacturer’s instructions. Antimicrobial susceptibility testing was additionally performed using the Kirby–Bauer disk diffusion method on Mueller–Hinton agar plates (Oxoid, UK) following Clinical and Laboratory Standards Institute (CLSI) guidelines (Clinical and Laboratory Standards Institute (CLSI), 2024). Bacterial suspensions equivalent to a 0.5 McFarland standard were prepared prior to inoculation.

Minimum inhibitory concentration (MIC) values generated by the VITEK® 2 system and phenotypic resistance profiles obtained from disk diffusion assays were comparatively evaluated to identify multidrug-resistant phenotypes across the analyzed isolates. Isolates exhibiting resistance to multiple antimicrobial classes were classified as multidrug-resistant (MDR). The selected isolate MRSA21-2025 demonstrated the broadest multidrug-resistant phenotype among the analyzed isolates and was therefore selected for long-read whole-genome sequencing and downstream comparative genomic analyses.

#### DNA extraction and whole-genome sequencing

Genomic DNA was extracted from the selected multidrug-resistant MRSA isolate (MRSA21-2025; SAMN57098905). Briefly, bacterial colonies grown on tryptic soy agar (TSA) plates were suspended in phosphate-buffered saline (PBS; pH 7.4) followed by centrifugation at 10,000 × g for 5 min. Genomic DNA extraction was performed using the QIAamp DNA Mini Kit (Qiagen, Hilden, Germany) according to the manufacturer’s instructions. Purified DNA was eluted in nuclease-free water and quantified using the Qubit™ dsDNA High Sensitivity (HS) Assay Kit (Thermo Fisher Scientific, United States), while DNA purity was evaluated using a NanoDrop™ 2000 spectrophotometer (Thermo Fisher Scientific, United States).

Oxford Nanopore sequencing libraries were prepared using the Native Barcoding Kit 24 V14 (Oxford Nanopore Technologies, UK) following the manufacturer’s protocol. Approximately 400 ng of genomic DNA was used for end-repair, barcode ligation, and library preparation. Sequencing was performed on a PromethION 2 Solo platform using R10.4.1 flow cells and MinKNOW software v24.06.5 with high-accuracy basecalling enabled (Wick et al., 2019).

Raw sequencing reads were quality-filtered prior to downstream analyses, and reads with quality scores below Q10 were excluded. Sequencing quality metrics and assembly statistics were subsequently evaluated during genome assembly and quality assessment analyses.

#### Genome assembly, annotation, and quality assessment

Raw Oxford Nanopore reads were subjected to *de novo* genome assembly using Flye v2.9 optimized for long-read assembly (Kolmogorov et al., 2019). The resulting draft assembly was subsequently polished using Medaka with the r1041_e82_400bps_sup_v5.0.0 model to improve consensus accuracy and reduce sequencing-associated errors.

Assembly quality metrics including genome size, contig number, N50 values, and sequencing depth were evaluated using QUAST (Gurevich et al., 2013). Sequencing statistics including total read count, total bases, read-length distribution, and mean read quality scores were additionally assessed to evaluate sequencing performance and assembly quality.

Genome annotation was performed using Prokka (Seemann, 2014), and annotation consistency across comparative genomes was standardized prior to downstream comparative genomic analyses. The assembled genome and associated sequencing data were deposited in the NCBI Sequence Read Archive (SRA) under accession number SRR37923555 (BioSample: SAMN57098905).

#### Genome annotation, typing, and resistome analysis

Genome annotation of the assembled MRSA21-2025 genome was performed using Prokka (Seemann, 2014) for standardized bacterial genome annotation prior to downstream comparative analyses. In silico multilocus sequence typing (MLST) was conducted based on the allelic profiles of seven housekeeping genes (*arcC, aroE, glpF, gmk, pta, tpi,* and *yqiL*) using the PubMLST *Staphylococcus aureus* scheme (Jolley et al., 2018). Spa typing was additionally performed to further characterize the lineage structure of the isolate.

Antimicrobial resistance determinants were identified using ABRicate (Seemann, 2016) by screening the assembled genome against multiple curated resistance databases including ResFinder (Bortolaia et al., 2020), CARD (Alcock et al., 2020), NCBI AMRFinderPlus (Feldgarden et al., 2021), and MEGARes (Doster et al., 2020). Default screening thresholds were applied using minimum nucleotide identity and coverage cutoffs of 90 and 80%, respectively. Detected resistance genes were subsequently categorized according to their associated antimicrobial classes and resistance mechanisms.

Virulence-associated genes were identified using ABRicate against the Virulence Factor Database (VFDB) (Chen et al., 2016). Detected virulence determinants were classified into functional categories including toxins, immune evasion-associated genes, proteases, adhesion-associated determinants, and leukocidin-related factors.

SCCmec typing was performed using SCCmecFinder v1.2 (Kaya et al., 2018) employing combined BLAST and k-mer-based approaches for *mec* and *ccr* complex identification. SCCmec classification thresholds included ≥90% nucleotide identity and minimum sequence coverage of 60%. Comparative SCCmec subtype metadata for publicly available ST6 genomes were additionally incorporated into downstream comparative phylogenomic analyses.

#### Comparative phylogenomics and pan-genome analysis

Comparative genomic analysis was performed to investigate the evolutionary placement of MRSA21-2025 within the global *Staphylococcus aureus* ST6 lineage. Publicly available genome sequences representing ST6 isolates were retrieved from the NCBI RefSeq database based on lineage relevance, assembly quality, assembly completeness, and metadata availability. Reference genomes included in the comparative dataset were selected to represent geographically diverse ST6-associated MRSA lineages and SCCmec backgrounds.

Core genome single nucleotide polymorphism (SNP) analysis was conducted using the Snippy pipeline v4.6.0 (Seemann, 2015) for variant calling and core genome alignment generation. Maximum-likelihood phylogenetic trees were reconstructed using IQ-TREE v2.2.0 (Nguyen et al., 2015) under the best-fit nucleotide substitution model. Branch support was assessed using ultrafast bootstrap approximation and SH-aLRT support analyses. Phylogenetic tree visualization and metadata annotation were performed using iTOL v5 (Letunic and Bork, 2021).

Comparative whole-genome similarity analyses were additionally performed using fastANI (Jain et al., 2018) to estimate pairwise average nucleotide identity (ANI) values among the analyzed ST6 genomes. Hierarchical ANI heatmaps were generated to visualize genome-wide similarity relationships and clustering patterns across the comparative dataset.

Pan-genome analysis was conducted using Panaroo v1.3.3 (Tonkin-Hill et al., 2020), which clusters orthologous genes while accounting for assembly and annotation inconsistencies. Genes were categorized into core, soft-core, shell, and cloud compartments to evaluate genomic conservation and accessory genome diversity across the analyzed ST6 lineage.

Core genome alignments generated using Panaroo strict mode were additionally analyzed using SNP-sites (Page et al., 2016) to identify variable positions and calculate pairwise SNP distance statistics across the comparative dataset. Summary statistics including minimum, maximum, mean, median, and standard deviation of pairwise core SNP distances were subsequently calculated for comparative evolutionary interpretation.

Comparative pan-genome clustering and accessory genome structure analyses were additionally evaluated using Roary (Page et al., 2015), while recombination-aware phylogenetic refinement was assessed using Gubbins (Croucher et al., 2015) to investigate lineage-specific diversification patterns among the analyzed ST6 genomes.

#### Mobile genetic element and prophage analysis

Mobile genetic elements (MGEs) associated with the assembled MRSA21-2025 genome were investigated using a combined analytical framework. Insertion sequence (IS) elements were identified and classified using ISEScan (Xie and Tang, 2017), enabling detection of IS families and prediction of transposition-associated regions across the assembled genome. Additional MGE screening was performed using MobileElementFinder (Johansson et al., 2021) to assess the distribution of resistance and virulence-associated mobile elements.

Prophage regions were identified using PHASTER (Arndt et al., 2016), while genomic islands were predicted using IslandViewer 4 (Bertelli et al., 2017). Plasmid-associated sequences were screened using PlasmidFinder (Carattoli et al., 2014). The genomic organization and chromosomal localization of identified MGEs were subsequently examined to evaluate their association with antimicrobial resistance determinants, SCCmec-associated regions, and virulence-associated loci.

Comparative analyses additionally assessed the distribution of IS families and prophage-associated regions within the broader ST6 comparative genomic dataset.

## Results

### Phenotypic characterization and isolate selection

A total of 50 clinical *Staphylococcus aureus* isolates recovered from endotracheal aspirate (ETA) specimens were screened using phenotypic antimicrobial susceptibility testing and VITEK® 2-based resistance profiling. Variable resistance patterns were observed across the analyzed isolates, with resistance detected against multiple antimicrobial classes including *β*-lactams, fluoroquinolones, aminoglycosides, macrolides, and tetracyclines.

Among the screened isolates, one isolate designated MRSA21-2025 (BioSample accession: SAMN57098905) exhibited the highest overall antimicrobial resistance profile and was therefore selected for long-read whole-genome sequencing and downstream comparative genomic analyses.

The selected isolate demonstrated resistance to multiple antimicrobial agents, including oxacillin, ciprofloxacin, erythromycin, gentamicin, and tetracycline. Comparative phenotypic resistance profiles for all screened isolates are summarized in Supplementary Table S1.

### Genome assembly and general features

Whole-genome sequencing of the multidrug-resistant MRSA isolate MRSA21-2025 (BioSample accession: SAMN57098905) generated a high-quality long-read genome assembly. *De novo* assembly of Oxford Nanopore reads produced a genome comprising 2,847,651 bp distributed across three contigs, with an average sequencing depth of approximately 267×. The largest contig measured 2,785,656 bp.

Genome assembly quality metrics generated by QUAST demonstrated high assembly continuity, with N50 and N90 values of 2,785,656 bp and L50/L90 values of 1. No ambiguous bases were detected within the assembled genome. BUSCO analysis using the bacteria_odb10 dataset identified complete recovery of conserved bacterial orthologs, with all detected BUSCOs present as single-copy genes.

**Figure 2:**
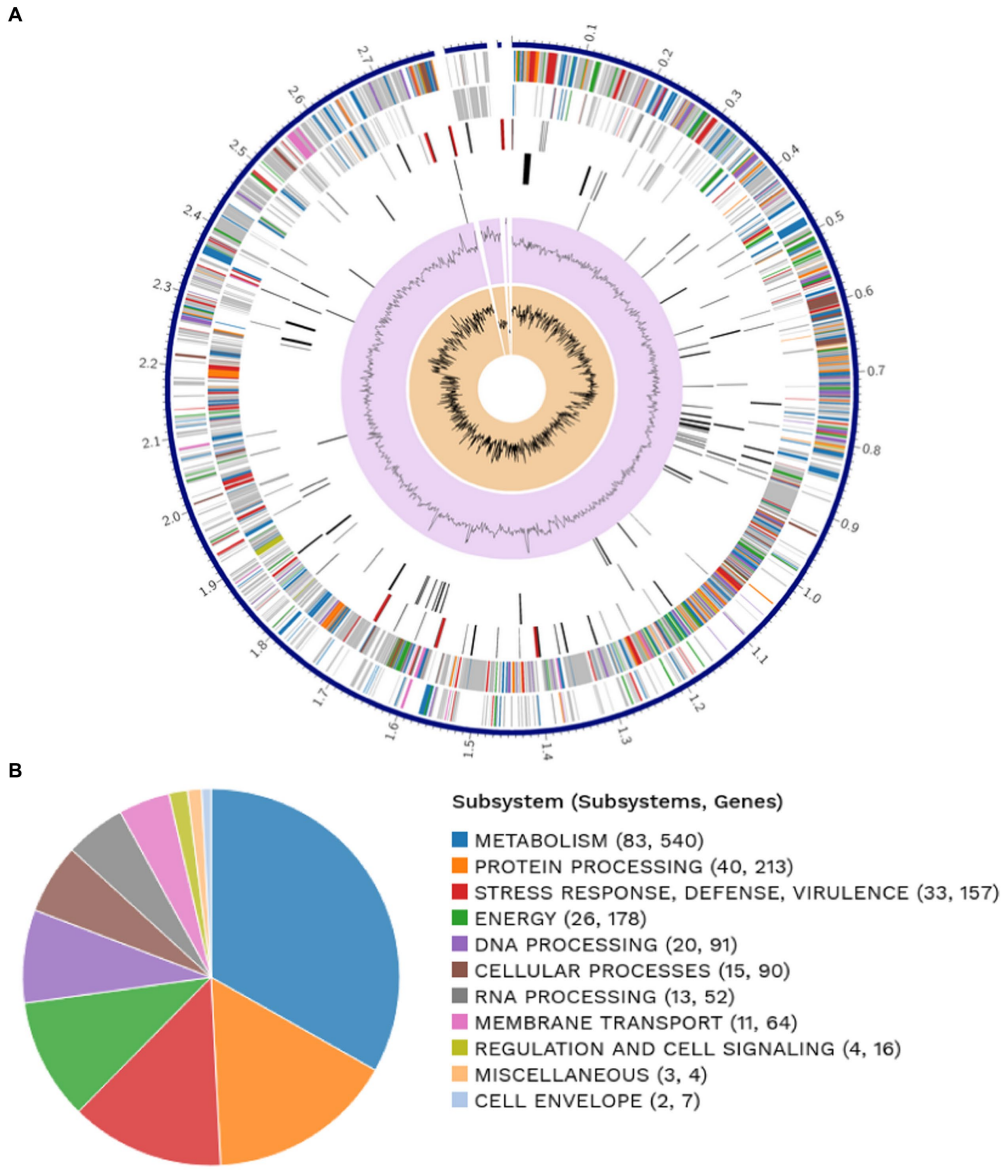
Genome structure and functional annotation of the MRSA21-2025 isolate. **(A)** Circular genome map illustrating the genomic architecture of MRSA21-2025. From outer to inner rings: contigs, coding DNA sequences (CDS) on the forward strand, CDS on the reverse strand, RNA genes, antimicrobial resistance-associated genes, virulence-associated genes, GC content, and GC skew. Functional subsystem classifications are represented by color coding across CDS regions. **(B)** Functional subsystem categorization of annotated genes identified in the assembled genome. The pie chart summarizes the distribution of genes across major biological processes including metabolism, protein processing, membrane transport, stress response and virulence, energy production, DNA processing, and additional cellular functions. The circular genome map and subsystem visualizations were generated using the BV-BRC (Bacterial and Viral Bioinformatics Resource Center) platform.

The assembled genome exhibited a GC content of 33.14% and contained 2,684 predicted protein-coding sequences, 33 tRNA genes, and 15 rRNA genes. Genome annotation and structural organization analyses are summarized in Table 1 and illustrated in Figure 2.

**Table 1:** Genome characteristics of MRSA21-2025 isolate.

Strain	Contigs	Genome length (bp)	GC content (%)	Protein-coding sequences (CDS)	tRNA genes	rRNA genes
MRSA21-2025	3	2,847,651	33.14	2,684	33	15

In silico multilocus sequence typing (MLST) assigned the isolate to sequence type ST6 based on the allelic profile of seven housekeeping genes (*arcC_12, aroE_4, glpF_1, gmk_4, pta_12, tpi_1,* and *yqiL_3*). All loci demonstrated 100% sequence identity and complete coverage relative to the reference alleles (Table 2).

**Table 2:** Multilocus sequence typing (MLST) allelic profile of the MRSA21-2025 isolate.

Locus	Identity (%)	Coverage (%)	Alignment length (bp)	Allele length (bp)	Assigned allele
*arcC*	100	100	456	456	*arcC*_12
*aroE*	100	100	456	456	*aroE*_4
*glpF*	100	100	465	465	*glpF*_1
*gmk*	100	100	417	417	*gmk*_4
*pta*	100	100	474	474	*pta*_12
*tpi*	100	100	402	402	*tpi*_1
*yqiL*	100	100	516	516	*yqiL*_3

Spa typing additionally classified the isolate as spa type t304.

The circular genome map and subsystem visualization were generated using the BV-BRC (Bacterial and Viral Bioinformatics Resource Center) platform (Olson et al., 2023).

### Molecular typing and SCCmec characterization

In silico molecular typing classified the MRSA21-2025 isolate as sequence type ST6 based on multilocus sequence typing (MLST) analysis of seven housekeeping genes (*arcC, aroE, glpF, gmk, pta, tpi,* and *yqiL*). All detected alleles demonstrated complete sequence identity and coverage relative to the corresponding reference alleles (Table 2).

Additional lineage characterization using spa typing identified the isolate as spa type t304. SCCmec analysis identified the presence of a SCCmec type IVa element carrying the *mecA* gene within the assembled chromosome. The SCCmec region contained a class B mec gene complex together with the *ccrA2* and *ccrB2* recombinase genes.

The SCCmec-associated region additionally contained *mecR1* and the insertion sequence IS1272. Comparative SCCmecFinder analysis demonstrated the highest similarity to SCCmec type IVa (2B) reference elements based on combined BLAST and k-mer-based classification metrics.

**Figure 3:**
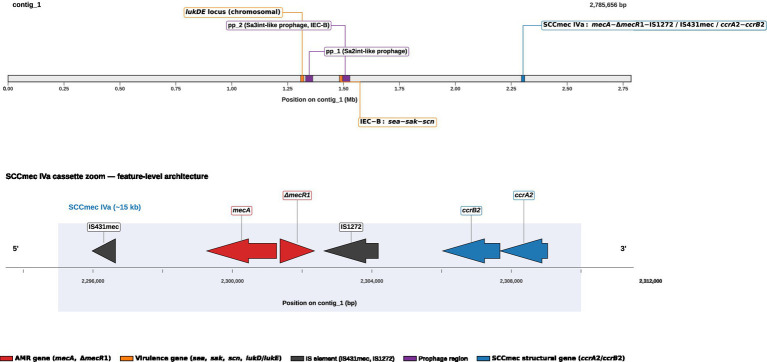
Chromosomal architecture and SCC*mec* IVa structural organization of MRSA21-2025. Chromosomal localization of major genomic features identified within contig_1 (2,785,656 bp) of the MRSA21-2025 genome assembly, including two prophage-associated regions (pp_1 and pp_2), the chromosomal *lukDE* locus, the IEC-associated prophage region carrying *sea*, *sak*, and *scn*, and the SCC*mec* IVa cassette containing *mecA*, Δ*mecR*1, IS1272/IS431*mec* insertion sequences, and the cassette chromosome recombinase genes *ccrA*2 and *ccrB*2. Expanded structural organization of the SCC*mec* IVa cassette (~15 kb) showing the arrangement and orientation of resistance-associated genes, insertion-sequence elements, and SCC*mec* recombinase genes within the chromosomal integration region. Chromosomal feature mapping and SCC*mec* structural visualization were generated using Proksee and manually refined for figure annotation and layout optimization.

Structural organization of the SCCmec element and its genomic context are illustrated in Figure 3.

Chromosomal feature mapping and SCCmec structural visualization were generated using Proksee and manually refined for figure annotation and layout optimization.

### Resistome profiling

Comprehensive resistome analysis of MRSA21-2025 identified multiple antimicrobial resistance-associated determinants distributed across several antimicrobial classes. Screening against CARD, ResFinder, NCBI AMRFinderPlus, and MEGARes databases identified genes associated with *β*-lactam resistance, multidrug efflux systems, tetracycline resistance, aminoglycoside resistance, and regulatory resistance-associated mechanisms.

The β-lactam resistance-associated region contained the *mecA* gene located within the SCCmec IVa cassette together with Δ*mecR1*. Additional resistance-associated determinants included *blaZ, tet38, norA, norC, mepA, mepR, arlS, arlR, mgrA, sdrM, lmrS,* and *kdpD*. Several detected loci were associated with multidrug efflux systems and regulatory resistance-associated pathways. Major resistance-associated determinants identified within the MRSA21-2025 genome and their predicted resistance mechanisms are summarized in Table 3.

**Table 3:** Integrated resistome profiling and CARD/RGI classification of major resistance-associated determinants identified in the MRSA21-2025 isolate.

Resistance-associated determinant	Sequence identity (%)	Genomic location	Associated antimicrobial class	Predicted resistance mechanism	CARD/RGI classification
*mecA*	100	contig_1	β-lactams	Altered penicillin-binding protein (PBP2a)	Strict
*norA*	100	contig_1	Fluoroquinolones	Efflux pump	Perfect
*norC*	100	contig_1	Fluoroquinolones	Efflux pump	Strict
*sdrM*	100	contig_1	Multidrug-associated/disinfectants	Efflux pump	Perfect
*lmrS*	100	contig_1	Macrolides/aminoglycosides	Efflux pump	Strict
*mepR*	100	contig_1	Tetracycline-associated	Regulatory-associated efflux modulation	Perfect
*mgrA*	100	contig_1	Multidrug-associated	Global transcriptional regulator	Perfect
*arlR*	100	contig_1	Regulatory-associated/fluoroquinolones	Two-component response regulator	Perfect
*arlS*	100	contig_1	Regulatory-associated/fluoroquinolones	Sensor histidine kinase	Perfect

Resistance-associated determinants were identified using integrated screening against CARD, ResFinder, AMRFinderPlus, and MEGARes databases. CARD/RGI classifications are shown according to Resistance Gene Identifier (RGI) prediction criteria.

*RGI, Resistance gene identifier.

*ARO, Antibiotic resistance ontology.

*Perfect and strict (i.e, with high confidence and probability).

CARD/RGI-based classification additionally identified multiple “Perfect” and “Strict” resistance-associated hits involving efflux systems, transcriptional regulators, and *β*-lactam resistance-associated determinants. The classification profiles and associated resistance ontology categories are summarized in Table 3.

Comparative resistome screening across the 51-genome ST6 dataset demonstrated conservation of several resistance-associated determinants among the analyzed genomes, while variability was observed in accessory loci including *blaZ* and selected efflux-associated genes.

**Figure 4:**
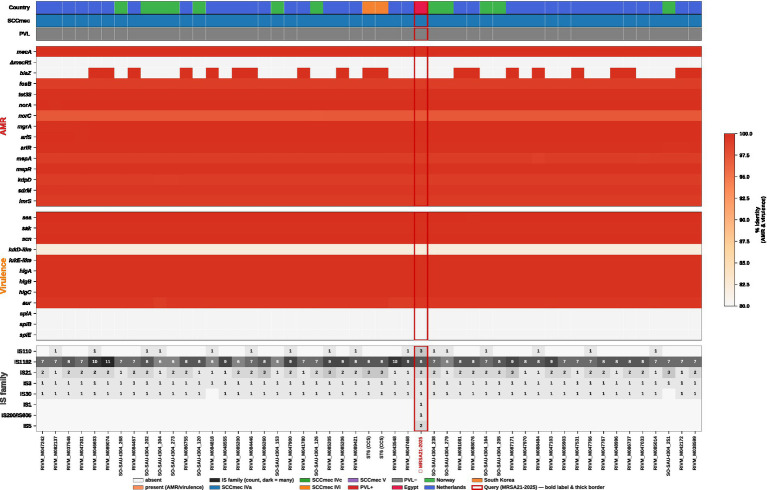
Comparative antimicrobial resistance, virulence-associated, and insertion-sequence family profiles across 51 ST6 *Staphylococcus aureus* genomes. Heatmap representation of antimicrobial resistance-associated genes, virulence-associated determinants, and insertion-sequence (IS) family distributions across the comparative ST6 genomic dataset. Columns represent individual genomes ordered according to the maximum-likelihood phylogenetic topology shown in Figure 5. Upper annotation tracks indicate country of origin, SCC*mec* subtype, and PVL status for each genome. The antimicrobial resistance-associated panel includes *mecA*, *mecR1*, *blaZ*, *fosB*, *tet38*, *norA*, *norC*, *mgrA*, *arlS*, *arlR*, *mepA*, *mepR*, *kdpD*, *sdrM*, and *lmrS*. The virulence-associated panel includes *sea*, *sak*, *scn*, *lukD*-like, *lukE*-like, *hlgA*, *hlgB*, *hlgC*, *aur*, *splA*, *splB*, and *splE*. Lower panels summarize the abundance and distribution of insertion-sequence families including IS*110*, IS*1182*, IS*21*, IS*3*, IS*30*, IS*1*, IS*200*/IS*605*, and IS*5*. The MRSA21-2025 query isolate is highlighted by a red-bordered column. Color intensity indicates relative presence and identity levels for resistance and virulence-associated loci, whereas grayscale intensity within IS-family panels corresponds to IS copy number abundance. Comparative heatmap visualization and metadata annotation were generated using custom Python-based scripts followed by graphical refinement in Adobe Illustrator.

The distribution of antimicrobial resistance-associated determinants across the comparative ST6 dataset is summarized in Figure 4. A hierarchical overview of resistance ontology classifications and associated resistance determinants identified in MRSA21-2025 is additionally presented in Supplementary Figure S2.

Comparative heatmap visualization and metadata annotation were generated using custom Python-based scripts and graphical post-processing in Adobe Illustrator.

### Virulence-associated genomic architecture

Virulence-associated gene screening of MRSA21-2025 identified multiple determinants associated with toxins, immune evasion, proteolytic activity, and leukocidin-associated functions. Screening against the Virulence Factor Database (VFDB) detected 96 virulence-associated genes distributed across several functional categories.

Toxin-associated loci included the gamma-hemolysin genes *hlgA, hlgB,* and *hlgC*, in addition to the leukocidin-associated genes *lukD* and *lukE*. Enterotoxin-associated screening identified the presence of the *sea* gene within a prophage-associated chromosomal region.

Immune evasion-associated determinants included *sak* and *scn*, both located within the IEC-associated prophage region integrated into the *β*-hemolysin locus. Protease-associated determinants included *aur, splA, splB,* and *splE*.

A leukocidin-associated prophage region additionally contained a *lukF-PV*-like homolog detected at approximately 82% sequence identity relative to reference PVL-associated sequences. Virulence-associated determinants identified in the MRSA21-2025 genome are summarized in Table 4.

**Table 4:** Major virulence-associated determinants identified in the MRSA21-2025 genome.

Virulence-associated determinant	Functional category	Genomic location	Predicted biological role
*hlgA*	Hemolysin-associated	contig_1	Gamma-hemolysin component A
*hlgB*	Hemolysin-associated	contig_1	Gamma-hemolysin component B
*hlgC*	Hemolysin-associated	contig_1	Gamma-hemolysin component C
*lukD*	Leukocidin-associated	contig_1	Leukocidin component D
*lukE*	Leukocidin-associated	contig_1	Leukocidin component E
*lukF-PV*-like homolog	Leukocidin-associated	prophage region (pp_1)	PVL-associated leukocidin homolog
*sea*	Enterotoxin-associated	prophage region (pp_2)	Staphylococcal enterotoxin A
*sak*	Immune evasion-associated	prophage region (pp_2)	Staphylokinase
*scn*	Immune evasion-associated	prophage region (pp_2)	Complement inhibitor
*aur*	Protease-associated	contig_1	Aureolysin metalloprotease
*splA*	Serine protease-associated	contig_1	Serine protease SplA
*splB*	Serine protease-associated	contig_1	Serine protease SplB
*splE*	Serine protease-associated	contig_1	Serine protease SplE

Comparative virulence-associated profiling across the 51-genome ST6 dataset demonstrated conservation of several core virulence-associated loci, while variability was observed in selected toxin-associated and prophage-associated determinants. The comparative distribution of major virulence-associated loci is illustrated in Figure 4.

### Mobile genetic elements and prophage characterization

Mobile genetic element analysis identified multiple insertion sequences (ISs), prophage-associated regions, and chromosomally integrated mobile elements within the MRSA21-2025 genome. ISEScan analysis detected 19 insertion sequence elements distributed across eight IS families, including IS110, IS1182, IS21, IS3, IS30, IS1, IS200/IS605, and IS5.

PlasmidFinder analysis did not identify plasmid-associated replicons within the assembled genome. Genomic island prediction identified multiple accessory genomic regions distributed across the chromosome, several of which overlapped with prophage-associated loci and resistance-associated regions.

PHASTER analysis identified two prophage-associated regions integrated within contig_1. The first prophage region (pp_1; approximately 33 kb) contained a *lukF-PV*-like homolog detected at approximately 82% sequence identity and was structurally consistent with a Sa2int-associated prophage organization. The second prophage region (pp_2; approximately 26 kb) was integrated within the *β*-hemolysin chromosomal region and contained the immune evasion-associated genes *sea, sak,* and *scn*, consistent with an IEC-B-associated prophage architecture.

Comparative analysis across the ST6 dataset demonstrated variability in IS-family abundance and prophage-associated loci among the analyzed genomes. Distribution patterns of IS families across the comparative dataset are summarized in Figure 4, while chromosomal organization of prophage-associated regions and SCCmec-associated loci is illustrated in Figure 3.

### Comparative phylogenomics

Core genome SNP-based phylogenetic analysis was performed using MRSA21-2025 together with 50 publicly available ST6 *Staphylococcus aureus* genomes representing geographically diverse isolates. Maximum-likelihood phylogenetic reconstruction based on core genome SNP alignments demonstrated clustering of the analyzed genomes into multiple lineage-associated branches within the ST6 population structure.

The MRSA21-2025 isolate clustered within a branch containing geographically related and SCCmec IV-associated genomes, including isolates originating from South Korea and the Netherlands. Several major phylogenetic clusters were supported by high bootstrap values, indicating stable lineage relationships across the analyzed dataset.

Comparative analysis additionally demonstrated geographic heterogeneity among the analyzed genomes, with multiple subclusters composed predominantly of isolates originating from the Netherlands and Norway. Branch length variability was observed across the phylogenetic topology, reflecting genomic divergence among the analyzed ST6 genomes.

**Figure 5:**
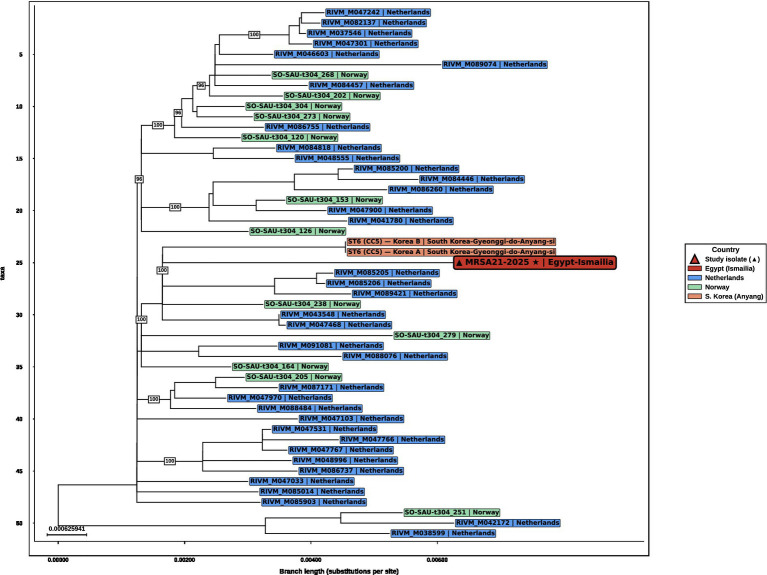
Core genome SNP-based maximum-likelihood phylogeny of 51 ST6 *Staphylococcus aureus* genomes including MRSA21-2025. Maximum-likelihood phylogenetic tree reconstructed from core genome single nucleotide polymorphism (SNP) alignments generated from 51 comparative ST6 *Staphylococcus aureus* genomes, including the Egyptian isolate MRSA21-2025 characterized in the present study. Core genome SNP alignments were generated using the Snippy pipeline, and phylogenetic reconstruction was performed using IQ-TREE under the best-fit nucleotide substitution model. Branch lengths represent nucleotide substitutions per site, and bootstrap support values ≥95 are indicated at major internal nodes. The MRSA21-2025 isolate is highlighted in red and marked with a triangle symbol. Colored labels indicate the geographic origin of each genome, including Egypt, the Netherlands, Norway, and South Korea. Tree visualization and metadata annotation were performed using iTOL v5.

The maximum-likelihood phylogenetic relationships and associated geographic metadata are illustrated in Figure 5.

### Whole-genome ANI and core genome SNP relationship analyses

Comparative whole-genome average nucleotide identity (ANI) analysis demonstrated a highly conserved genomic background across the analyzed ST6-*Staphylococcus aureus* dataset. Pairwise ANI values ranged from 99.9246 to 99.9793%, with a mean ANI of 99.9542% ± 0.0112%, supporting close evolutionary relatedness among the analyzed genomes despite geographic diversity.

The highest ANI value relative to MRSA21-2025 was observed for the Dutch isolate RIVM_M091081 (99.9793%), while the lowest ANI value was identified for the Norwegian isolate SO-SAU-t304_251 (99.9246%). Comparative ANI clustering patterns demonstrated overall conservation of the ST6-IVa genomic backbone across geographically distributed isolates from Europe, Asia, and Egypt.

Core genome SNP distance analysis identified 2,565 variable sites within the concatenated Panaroo core genome alignment, including 759 parsimony-informative positions and 1,806 singleton sites. Pairwise core genome SNP distances between MRSA21-2025 and the comparative reference genomes ranged from 188 to 367 SNPs, while pairwise distances among reference genomes ranged from 0 to 300 SNPs. Detailed pairwise core genome SNP distance summary statistics across the comparative ST6 dataset are provided in Supplementary Table S4.

The closest phylogenomic relatives of MRSA21-2025 included Dutch and Norwegian ST6-IVa isolates, including RIVM_M043548 and SO-SAU-t304_238, both differing by 188 core genome SNPs. These findings support phylogenetic conservation within the globally distributed ST6-IVa lineage while additionally indicating moderate geographic diversification. Core phylogenomic framework statistics and comparative genomic summary metrics for the analyzed ST6 dataset are presented in Table 5.

**Table 5:** Core genome phylogenomic framework and comparative genomic statistics of the ST6 dataset.

Parameter	Value
Total genomes analyzed	51
Core genes (Panaroo strict)	2,495
Total pan-genome size	2,680 gene clusters
Concatenated core alignment length	2,321,111 bp
Total variable sites (snp-sites)	2,565
Parsimony-informative sites	759
Singleton sites	1,806
Phylogenetic reconstruction method	Maximum-likelihood
IQ-TREE substitution model	GTR + ASC
Number of taxa included	51
Mean pairwise core SNP distance (full dataset)	151.2 ± 46.1
Median pairwise core SNP distance (full dataset)	145
Core SNP distance range (full dataset)	0–367
ANI range across comparative genomes	99.9246–99.9793%
Mean ANI ± SD	99.9542% ± 0.0112%

**Figure 6:**
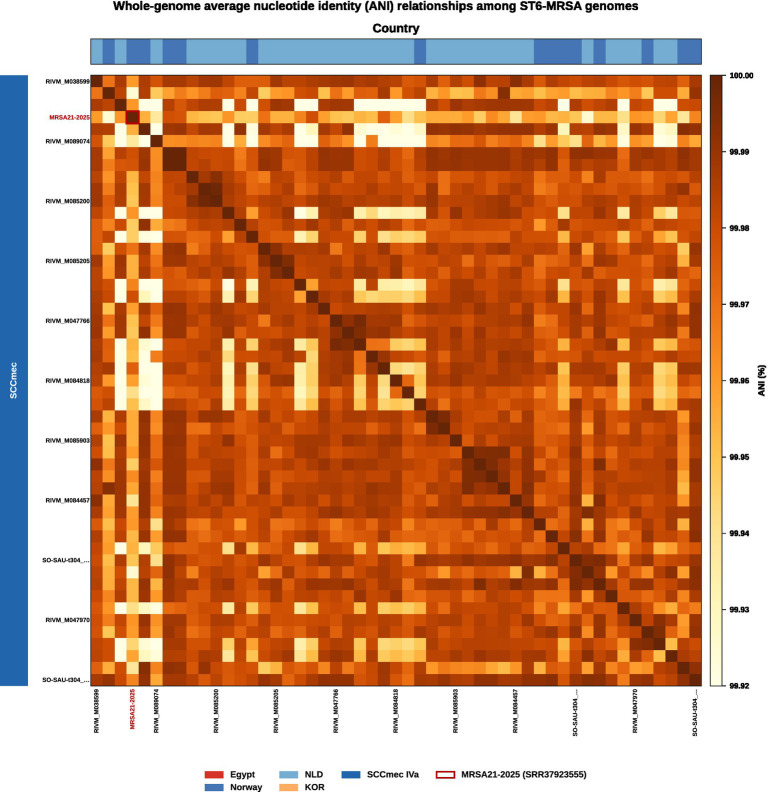
Whole-genome average nucleotide identity (ANI) relationships among comparative ST6-MRSA genomes. Hierarchically clustered heatmap showing pairwise whole-genome average nucleotide identity (ANI) values among 51 comparative ST6-MRSA genomes, including MRSA21-2025 and 50 publicly available reference genomes. ANI values were calculated using fastANI to evaluate whole-genome nucleotide conservation across the comparative dataset. Color intensity corresponds to ANI percentage values ranging from 99.92 to 100%, with darker colors indicating higher genomic similarity. Metadata annotation bars indicate country of origin and SCC*mec* subtype distribution across the analyzed genomes. The Egyptian isolate clustered within the ST6-t304-SCC*mec* IVa lineage and demonstrated high genomic similarity to multiple European reference genomes. Only every fifth genome label is displayed for visualization clarity, whereas the complete genome list and clustering order are provided in Supplementary Table S2. Pairwise ANI calculations were performed using fastANI v1.34, and hierarchical heatmap visualization was generated in Python using seaborn, matplotlib, and scipy.cluster.hierarchy.

**Figure 7:**
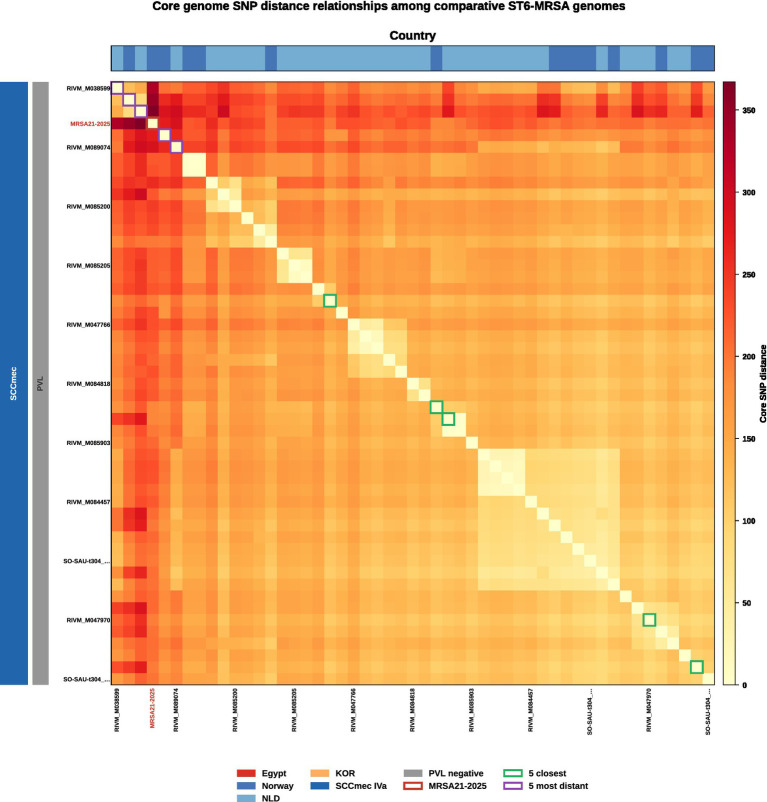
Core genome SNP distance relationships among comparative ST6-MRSA genomes. Heatmap showing pairwise core genome SNP distances among 51 comparative ST6-MRSA genomes, including MRSA21-2025 and 50 publicly available reference genomes. Core genome SNPs were identified from Panaroo strict-mode core genome alignments and pairwise SNP distances were calculated from concatenated variable positions. Lighter colors indicate lower SNP distances and closer phylogenomic relatedness, whereas darker colors indicate greater genomic divergence. Metadata annotation bars indicate country of origin, SCC*mec* subtype, and PVL status across the comparative dataset. Green boxes highlight the five genomes most closely related to MRSA21-2025, whereas purple boxes indicate the five most genetically distant genomes. Only every fifth genome label is displayed for visualization clarity, while the complete clustering order is provided in Supplementary Table S3. Pairwise core genome SNP distance analyses were generated using Panaroo v1.3.3 and SNP-sites and visualized in Python using seaborn, matplotlib, and scipy.cluster.hierarchy.

Comparative ANI relationships and core genome SNP distance distributions across the analyzed ST6 dataset are illustrated in Figures 6, 7, respectively. Complete comparative genome metadata and ranked pairwise SNP distance statistics are provided in Supplementary Tables S2, S3.

Core genome reconstruction and pan-genome analyses were performed using Panaroo. Core SNP alignments were generated using Snippy and analyzed using IQ-TREE under the GTR + ASC substitution model. Whole-genome average nucleotide identity (ANI) comparisons were calculated using fastANI.

Pairwise ANI values were calculated using fastANI v1.34 and visualized as a hierarchically clustered heatmap generated in Python using the seaborn, matplotlib, and scipy.cluster.hierarchy libraries.

### Pan-genome analysis

Pan-genome analysis of MRSA21-2025 together with 50 publicly available ST6 *Staphylococcus aureus* genomes identified a total pan-genome consisting of 2,680 gene clusters. Among these, 2,495 genes were classified as core genes shared across the analyzed dataset, while 34 genes were categorized as soft-core genes. Accessory genome analysis identified 118 shell genes and 33 cloud genes variably distributed among the analyzed genomes.

The distribution of core and accessory genome compartments demonstrated conservation of the majority of housekeeping and essential chromosomal loci across the analyzed ST6 lineage, while variability was observed within accessory genomic regions associated with mobile genetic elements and prophage-associated loci.

**Figure 8:**
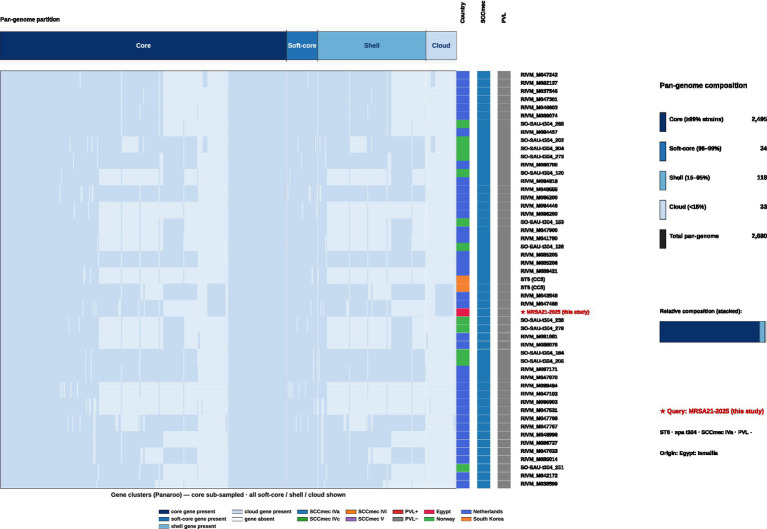
Comparative pan-genome structure of 51 ST6 *Staphylococcus aureus* genomes generated using Panaroo. Presence–absence matrix of orthologous gene clusters across the comparative ST6 genomic dataset, including the MRSA21-2025 query isolate. Columns represent individual genomes ordered according to the phylogenetic topology shown in Figure 5, whereas rows represent clustered gene families identified by Panaroo. Colored blocks indicate gene presence and white regions indicate gene absence. Gene clusters were categorized into core, soft-core, shell, and cloud compartments according to their distribution frequencies across the analyzed genomes. The comparative dataset consisted of 2,495 core genes, 34 soft-core genes, 118 shell genes, and 33 cloud genes, resulting in a total pan-genome size of 2,680 gene clusters. Upper metadata tracks indicate country of origin, SCC*mec* subtype, and PVL status for each genome. The MRSA21-2025 isolate is highlighted in red within the comparative dataset. The pan-genome presence–absence matrix and comparative visualization were generated using Panaroo and custom Python-based visualization scripts.

Presence–absence matrix analysis demonstrated heterogeneity in accessory genome composition among the analyzed ST6 genomes. Comparative pan-genome structure and accessory gene distribution patterns are illustrated in Figure 8.

## Discussion

Comparative phylogenomic analysis demonstrated that the Egyptian MRSA21-2025 isolate belongs to a globally distributed ST6-t304-SCCmec IVa lineage characterized by remarkable core genome conservation despite substantial geographic separation. The observed phylogenomic clustering of MRSA21-2025 with Northern European isolates, particularly genomes originating from the Netherlands and Norway, suggests that the analyzed isolate represents part of an internationally disseminated ST6 genomic backbone rather than an independently evolving local Egyptian clone (Olson et al., 2023; Bartels et al., 2021). Importantly, although whole-genome ANI values remained exceptionally high across the comparative dataset (>99.92%), core genome SNP analysis identified measurable lineage diversification ranging from 188 to 367 SNPs relative to publicly available reference genomes. This degree of divergence likely reflects progressive microevolutionary adaptation and long-term lineage circulation rather than recent short-term clonal expansion.

The high prevalence of multidrug-resistant phenotypes identified among the analyzed clinical isolates is consistent with previous reports describing increasing MRSA-associated antimicrobial resistance rates across Egypt and neighboring Middle Eastern and North African regions. Among the 50 analyzed isolates, 46 isolates (92%) exhibited multidrug-resistant phenotypes, highlighting the substantial local burden of antimicrobial resistance within the investigated clinical population. Importantly, although the comparative phylogenomic analysis demonstrated that MRSA21-2025 belongs to a globally conserved ST6-t304-SCCmec IVa lineage, the observed clustering pattern did not support strict regional segregation. Instead, the Egyptian isolate exhibited close evolutionary relatedness to European clinical genomes, particularly isolates originating from the Netherlands and Norway, suggesting potential historical interregional dissemination and long-term circulation of this lineage across geographically distinct healthcare-associated and community-associated settings. These findings collectively support the growing recognition that successful MRSA lineages are increasingly shaped by international transmission dynamics, population mobility, healthcare-associated dissemination, and adaptive chromosomal evolution rather than isolated local emergence alone.

The identification of a globally related ST6-IVa lineage within Egypt has important epidemiological implications because genomic investigations focusing on ST6-t304-SCCmec IVa MRSA remain extremely limited within the Middle East and North Africa region. Previous studies describing ST6-IVa MRSA have largely originated from Northern Europe, where this lineage has emerged as an increasingly successful community-associated and healthcare-associated clone (Bartels et al., 2021; Petersen et al., 2021; Kløve et al., 2022; Sainmaa et al., 2023). Accordingly, the present study provides important regional genomic epidemiology data supporting potential interregional dissemination of ST6-associated MRSA lineages beyond previously recognized geographic boundaries. The close evolutionary relationship observed between the Egyptian isolate and European genomes may reflect historical international transmission events, population mobility, healthcare-associated dissemination, or underrecognized circulation of ST6 lineages within the broader Mediterranean and Middle Eastern regions.

A major finding of the present investigation was the coexistence of extensive chromosomally encoded multidrug resistance-associated determinants together with structurally conserved SCCmec IVa architecture in the absence of detectable plasmid replicons. This genomic configuration suggests that the adaptive success of the analyzed lineage is driven predominantly by chromosomal evolution, recombination-associated diversification, and mobile chromosomal elements rather than plasmid-mediated acquisition alone. Such chromosomally stabilized resistance architectures may provide enhanced long-term evolutionary stability while simultaneously preserving the capacity for acquisition and reshuffling of accessory virulence-associated and resistance-associated loci through insertion-sequence-mediated and prophage-mediated genomic plasticity. The observed co-localization of SCCmec-associated regions with multiple insertion-sequence elements and prophage-associated loci further supports ongoing chromosomal remodeling and adaptive genome restructuring within the analyzed isolate.

The SCCmec IVa cassette identified in MRSA21-2025 exhibited structural organization highly similar to globally disseminated ST6-IVa lineages previously described in Europe (Bartels et al., 2021; Gaber et al., 2024). SCCmec IV-associated MRSA clones are frequently linked to enhanced epidemiological dissemination because of the comparatively smaller size and reduced biological fitness cost of SCCmec IV elements relative to larger SCCmec architectures. The coexistence of SCCmec IVa with multiple multidrug efflux-associated determinants, global regulatory genes, adhesion-associated loci, and immune evasion-associated prophage genes may therefore represent an evolutionarily optimized genomic configuration favoring persistence, transmission, host adaptation, and multidrug survival under sustained antimicrobial selection pressure.

Pan-genome reconstruction additionally demonstrated that diversification within the analyzed ST6 population occurred despite strong conservation of housekeeping genomic regions. More than 93% of all identified gene clusters belonged to the conserved core genome compartment, whereas shell and cloud genes were predominantly associated with mobile genetic elements, prophage-associated regions, and adaptive accessory loci. These findings strongly support the concept that successful MRSA lineages evolve primarily through acquisition, stabilization, and genomic reshuffling of accessory elements while maintaining a highly stable core chromosomal backbone. The accessory genome variability identified in the present study therefore likely reflects ongoing adaptive evolution mediated by horizontal gene transfer, recombination, prophage acquisition, and insertion-sequence activity.

An additional major strength of the present study was the application of long-read Oxford Nanopore sequencing for high-resolution reconstruction of structurally complex chromosomal regions. Long-read assembly enabled near-complete resolution of SCCmec-associated loci, insertion-sequence distributions, prophage-associated regions, and chromosomal virulence-associated architectures that are frequently fragmented or incompletely resolved using short-read sequencing approaches. The ability to reconstruct these highly repetitive genomic regions substantially improved interpretation of genomic context, structural organization, and potential mechanisms underlying dissemination of resistance-associated and virulence-associated determinants. Importantly, this structural resolution provided clearer insight into chromosomal organization of the ST6-IVa lineage than would typically be achievable using fragmented draft assemblies.

The identification of IEC-associated prophage genes including *sea*, *sak*, and *scn* together with leukocidin-associated loci further highlights the contribution of prophage-mediated horizontal transfer to pathogenic evolution and host adaptation within ST6-MRSA populations. Previous studies have demonstrated that immune evasion cluster-associated prophages contribute substantially to colonization efficiency and persistence within human-associated *Staphylococcus aureus* lineages. The coexistence of immune evasion determinants, toxin-associated loci, multidrug resistance-associated mechanisms, and structurally conserved SCCmec IVa architecture within a globally related ST6 background may therefore represent a highly adaptive genomic configuration facilitating both persistence and transmission in healthcare-associated and community-associated settings.

Collectively, the present study demonstrates that integration of long-read sequencing, comparative phylogenomics, pan-genome reconstruction, mobilome characterization, and structural SCCmec analysis provides a powerful framework for high-resolution genomic surveillance of emerging MRSA lineages. The generated genomic dataset provides important insight into the evolutionary dynamics, mobilome composition, chromosomal resistance architecture, and international phylogenomic relationships of an Egyptian ST6-IVa MRSA lineage. These findings further emphasize the importance of implementing genome-based surveillance frameworks capable of early detection and monitoring of emerging multidrug-resistant MRSA clones with regional and global dissemination potential.

Although comparative phylogenomic analysis demonstrated close relatedness between MRSA21-2025 and publicly available international ST6-IVa genomes, the present study was based on a single representative isolate and therefore does not permit direct inference regarding transmission dynamics, prevalence, or epidemiological dominance of this lineage within Egypt. Broader longitudinal genomic surveillance involving larger isolate collections will be required to clarify the regional epidemiology and dissemination patterns of ST6-associated MRSA lineages.

## Conclusion

Whole-genome sequencing and high-resolution comparative genomic analysis of the multidrug-resistant MRSA isolate MRSA21-2025 revealed a complex chromosomal genomic architecture characterized by the coexistence of the *mecA* gene, multiple multidrug efflux-associated resistance determinants, structurally conserved SCCmec IVa elements, diverse mobile genetic elements, and a broad repertoire of virulence-associated loci including adhesion-associated, toxin-associated, leukocidin-associated, and immune evasion-associated genes. Comparative phylogenomics positioned the isolate within a globally distributed ST6-t304-SCCmec IVa lineage exhibiting remarkable core genome conservation despite measurable geographic and accessory genome diversification.

Comprehensive genomic reconstruction further demonstrated the important contribution of insertion sequences, prophage-associated regions, chromosomal mobile elements, and accessory resistance-associated loci to the adaptive potential and evolutionary stability of the analyzed lineage. The observed coexistence of multidrug resistance-associated determinants, chromosomal genomic mobility, immune evasion-associated prophages, and multifactorial virulence architectures highlights the evolutionary success and clinical significance of this emerging ST6-IVa MRSA lineage.

Importantly, comprehensive long-read comparative genomic investigations focusing on ST6-t304-SCCmec IVa MRSA isolates remain extremely limited within Egypt and the broader Middle East and North Africa region. Accordingly, the present study provides valuable regional genomic epidemiology data describing the evolutionary dynamics, mobilome composition, structural SCCmec organization, accessory genome diversification, and international phylogenomic relationships of an Egyptian ST6-IVa lineage. Integration of long-read sequencing, comparative phylogenomics, pan-genome reconstruction, mobilome characterization, and genome-based surveillance therefore represents a powerful framework for monitoring the emergence, adaptation, and dissemination of clinically relevant multidrug-resistant MRSA clones with regional and global public health significance.

## Limitation

Despite these findings, the present study has several limitations. The analysis was based on a single representative isolate and therefore may not fully reflect the genomic diversity and epidemiological distribution of MRSA lineages circulating within Egypt. In addition, the study relied primarily on genome-based predictions without experimental functional validation of the identified resistance-associated and virulence-associated determinants. Comparative analyses were also restricted to publicly available ST6 genomes with adequate assembly quality and metadata availability. Future studies incorporating larger longitudinal isolate collections together with functional validation experiments would further strengthen understanding of the evolutionary dynamics, genomic diversity, and dissemination patterns of ST6-associated MRSA lineages.

## Data Availability

The sequencing data generated in this study are publicly available in the NCBI Sequence Read Archive (SRA) under accession number SRR37923555 (BioSample: SAMN57098905, *Staphylococcus aureus* MRSA21-2025). All relevant data supporting the findings of this study are included within the article and its Supplementary materials.

## Glossary

AMR: Antimicrobial resistance
ANI: Average nucleotide identity
AST: Antimicrobial susceptibility testing
BMD: Broth microdilution
BUSCO: Benchmarking universal single-copy orthologs
CARD: Comprehensive antibiotic resistance database
CCR: Cassette chromosome recombinase
CDS: Coding DNA sequence
ETA: Endotracheal aspirate
GC: Guanine–cytosine content
HAC: High-accuracy basecalling
ICU: Intensive care unit
iTOL: Interactive tree of life
k-mer: k-length subsequence
MGEs: Mobile genetic elements
MIC: Minimum inhibitory concentration
MLST: Multilocus sequence typing
MRSA: Methicillin-resistant *Staphylococcus aureus*
ONT: Oxford nanopore technologies
PBS: Phosphate-buffered saline
PVL: Panton–valentine leukocidin
QC: Quality control
QUAST: Quality assessment tool for genome assemblies
RASTtk: Rapid annotation using subsystem technology toolkit
RGI: Resistance gene identifier
SCCmec: Staphylococcal cassette chromosome mec
SNP: Single nucleotide polymorphism
ST: Sequence type
TSA: Tryptic soy agar
VFDB: Virulence factor database
WGS: Whole-genome sequencing
