# Supplementary material for: Comparative phylogenomic and long-read genomic characterization of an Egyptian ST6-MRSA-IVa clinical isolate within a globally conserved multidrug-resistant lineage
Source: Front Microbiol. 2026 Jun 8;17:1855574. doi: 10.3389/fmicb.2026.1855574 (PMC13284069; doi:10.3389/fmicb.2026.1855574)
Supplement: Supplementary file 1 [file Table_1.DOCX]

## **Supplementary** Table S1. Comparative phenotypic antimicrobial susceptibility profiles of 50 clinical Staphylococcus aureus isolates recovered from endotracheal aspirate (ETA) specimens.

| Sample ID | Isolate Code | Hospital | ST | FOX | OXA | CIP | LEV | GEN | ERY | CLI | VAN | Profile |
| --- | --- | --- | --- | --- | --- | --- | --- | --- | --- | --- | --- | --- |
| 1 | MRSA-01 | Kasr Al-Ainy Hospital | NA | R | R | R | R | S | R | R | S | MDR |
| 2 | MRSA-02 | Ain Shams University Hospital | NA | R | R | S | R | R | R | S | S | MDR |
| 3 | MRSA-03 | El Demerdash Hospital | NA | R | R | R | R | R | R | R | S | MDR |
| 4 | MRSA-04 | Mansoura University Hospital | NA | R | R | R | S | S | R | R | S | MDR |
| 5 | **MRSA21-2025** | **Military Medical Academy Hospital** | **ST6** | **R** | **R** | **R** | **R** | **R** | **R** | **R** | **S** | **MDR** |
| 6 | MRSA-06 | Alexandria Main University Hospital | NA | R | R | R | R | R | S | R | S | MDR |
| 7 | MRSA-07 | Tanta University Hospital | NA | R | R | R | R | R | R | R | S | MDR |
| 8 | MRSA-08 | Zagazig University Hospital | NA | R | R | S | R | R | R | R | S | MDR |
| 9 | MRSA-09 | Benha University Hospital | NA | R | R | R | R | S | R | R | S | MDR |
| 10 | MRSA-10 | Assiut University Hospital | NA | R | R | R | R | R | R | S | S | MDR |
| 11 | MRSA-11 | Sohag University Hospital | NA | R | R | R | S | R | R | R | S | MDR |
| 12 | MRSA-12 | Minia University Hospital | NA | R | R | R | R | R | R | R | S | MDR |
| 13 | MRSA-13 | Fayoum University Hospital | NA | R | R | R | R | R | S | R | S | MDR |
| 14 | MRSA-14 | Beni-Suef University Hospital | NA | R | R | S | R | R | R | R | S | MDR |
| 15 | MRSA-15 | Suez Canal University Hospital | NA | R | R | R | R | R | R | R | S | MDR |
| 16 | MRSA-16 | Port Said General Hospital | NA | S | S | S | S | S | S | S | S | Non-MDR |
| 17 | MRSA-17 | Ismailia Fever Hospital | NA | R | R | R | R | R | R | R | S | MDR |
| 18 | MRSA-18 | Damietta General Hospital | NA | R | R | R | R | S | R | R | S | MDR |
| 19 | MRSA-19 | Kafr El-Sheikh General Hospital | NA | R | R | R | S | R | R | R | S | MDR |
| 20 | MRSA-20 | Damanhour Medical Institute | NA | R | R | R | R | R | R | R | S | MDR |
| 21 | MRSA-21 | Mahalla Chest Hospital | NA | R | R | S | R | R | R | R | S | MDR |
| 22 | MRSA-22 | Banha Teaching Hospital | NA | R | R | R | R | R | S | R | S | MDR |
| 23 | MRSA-23 | El Salam International Hospital | NA | R | R | R | R | R | R | R | S | MDR |
| 24 | MRSA-24 | Nasser Institute Hospital | NA | R | R | R | S | S | R | R | S | MDR |
| 25 | MRSA-25 | Dar Al Fouad Hospital | NA | R | R | R | R | R | R | R | S | MDR |
| 26 | MRSA-26 | Air Force Specialized Hospital | NA | R | R | R | R | R | R | S | S | MDR |
| 27 | MRSA-27 | Police Authority Hospital | NA | R | R | R | R | S | R | R | S | MDR |
| 28 | MRSA-28 | El Agouza Hospital | NA | R | R | S | R | R | R | R | S | MDR |
| 29 | MRSA-29 | El Helal Hospital | NA | R | R | R | R | R | R | R | S | MDR |
| 30 | MRSA-30 | Shebin El-Kom Teaching Hospital | NA | R | R | R | R | R | S | R | S | MDR |
| 31 | MRSA-31 | Menoufia University Hospital | NA | R | R | R | S | R | R | R | S | MDR |
| 32 | MRSA-32 | Qena General Hospital | NA | R | R | R | R | R | R | R | S | MDR |
| 33 | MRSA-33 | Luxor International Hospital | NA | R | R | S | R | R | R | R | S | MDR |
| 34 | MRSA-34 | Aswan University Hospital | NA | R | R | R | R | R | S | R | S | MDR |
| 35 | MRSA-35 | Hurghada General Hospital | NA | R | R | R | R | R | R | R | S | MDR |
| 36 | MRSA-36 | Sharm El-Sheikh International Hospital | NA | R | R | R | R | S | R | R | S | MDR |
| 37 | MRSA-37 | Matrouh General Hospital | NA | R | R | R | S | R | R | R | S | MDR |
| 38 | MRSA-38 | New Cairo Hospital | NA | R | R | R | R | R | R | R | S | MDR |
| 39 | MRSA-39 | El Obour Specialized Hospital | NA | R | R | S | R | R | R | R | S | MDR |
| 40 | MRSA-40 | Gamal Abdel Nasser Hospital | NA | R | R | R | R | R | S | R | S | MDR |
| 41 | MRSA-41 | Helwan General Hospital | NA | R | R | R | R | R | R | R | S | MDR |
| 42 | MRSA-42 | October 6 University Hospital | NA | R | R | R | S | S | R | R | S | MDR |
| 43 | MRSA-43 | Misr International Hospital | NA | R | R | R | R | R | R | R | S | MDR |
| 44 | MRSA-44 | National Cancer Institute Hospital | NA | R | R | R | R | R | S | R | S | MDR |
| 45 | MRSA-45 | El Fayrouz Medical Complex | NA | S | S | S | S | S | S | S | S | Non-MDR |
| 46 | MRSA-46 | Badr University Hospital | NA | R | R | R | R | R | R | R | S | MDR |
| 47 | MRSA-47 | Cleopatra Hospital | NA | R | R | R | S | R | R | R | S | MDR |
| 48 | MRSA-48 | Saudi German Hospital Cairo | NA | R | R | R | R | R | R | R | S | MDR |
| 49 | MRSA-49 | Al Mokattam Health Center | NA | S | S | S | S | S | S | S | S | Non-MDR |
| 50 | MRSA-50 | El Nasr Specialized Hospital | NA | S | S | S | S | S | S | S | S | Non-MDR |

## **Supplementary Table S2. Genomes included in the comparative ANI heatmap and phylogenomic analysis of ST6-*Staphylococcus aureus* isolates**

A total of 51 ST6-*Staphylococcus aureus* genomes were included in the comparative ANI and phylogenomic analyses, comprising one Egyptian study isolate and 50 publicly available reference genomes. Genome order corresponds directly to the clustered heatmap order shown in Figure 2. For visualization clarity, only the study isolate and every fifth genome in the clustered dataset were labelled directly on the heatmap axes.

| Order | Strain name | Assembly accession | Country of origin | ST | spa type | SCCmec subtype | PVL status | Labelled in Figure 2 |
| --- | --- | --- | --- | --- | --- | --- | --- | --- |
| 1 | RIVM_M038599 | GCA_027706345.1 | Netherlands | 6 | t304 | IVa | − | Yes |
| 2 | SO-SAU-t304_251 | GCA_018983475.1 | Norway | 6 | t304 | IVa | − | No |
| 3 | RIVM_M042172 | GCA_027818655.1 | Netherlands | 6 | t304 | IVa | − | No |
| 4 | MRSA21-2025 (this study) | SRR37923555 | Egypt: Ismailia | 6 | t304 | IVa | − | Yes |
| 5 | SO-SAU-t304_279 | GCA_018983245.1 | Norway | 6 | t304 | IVa | − | No |
| 6 | RIVM_M089074 | GCA_027759845.1 | Netherlands | 6 | t304 | IVa | − | Yes |
| 7 | ST6 (CC5) — Korea A | GCA_054559635.1 | South Korea | 6 | t304 | IVa | − | No |
| 8 | ST6 (CC5) — Korea B | GCA_054559695.1 | South Korea | 6 | t304 | IVa | − | No |
| 9 | RIVM_M086260 | GCA_027652965.1 | Netherlands | 6 | t4403 | IVa | − | No |
| 10 | RIVM_M084446 | GCA_027632305.1 | Netherlands | 6 | t304 | IVa | − | No |
| 11 | RIVM_M085200 | GCA_027648325.1 | Netherlands | 6 | t304 | IVa | − | Yes |
| 12 | RIVM_M041780 | GCA_027824535.1 | Netherlands | 6 | t304 | IVa | − | No |
| 13 | RIVM_M047900 | GCA_027718925.1 | Netherlands | 6 | t304 | IVa | − | No |
| 14 | SO-SAU-t304_153 | GCA_018984155.1 | Norway | 6 | t304 | IVa | − | No |
| 15 | RIVM_M089421 | GCA_027772605.1 | Netherlands | 6 | t304 | IVa | − | No |
| 16 | RIVM_M085205 | GCA_027630665.1 | Netherlands | 6 | t304 | IVa | − | Yes |
| 17 | RIVM_M085206 | GCA_027648385.1 | Netherlands | 6 | t304 | IVa | − | No |
| 18 | RIVM_M088076 | GCA_027759005.1 | Netherlands | 6 | t304 | IVa | − | No |
| 19 | RIVM_M091081 | GCA_027771485.1 | Netherlands | 6 | t304 | IVa | − | No |
| 20 | RIVM_M047103 | GCA_042904085.1 | Netherlands | 6 | t304 | IVa | − | No |
| 21 | RIVM_M047766 | GCA_042932485.1 | Netherlands | 6 | t304 | IVa | − | Yes |
| 22 | RIVM_M047767 | GCA_042932505.1 | Netherlands | 6 | t304 | IVa | − | No |
| 23 | RIVM_M047531 | GCA_042885885.1 | Netherlands | 6 | t304 | IVa | − | No |
| 24 | RIVM_M048996 | GCA_027720465.1 | Netherlands | 6 | t304 | IVa | − | No |
| 25 | RIVM_M086737 | GCA_027768905.1 | Netherlands | 6 | t304 | IVa | − | No |
| 26 | RIVM_M084818 | GCA_027632845.1 | Netherlands | 6 | t304 | IVa | − | Yes |
| 27 | RIVM_M048555 | GCA_027642105.1 | Netherlands | 6 | t304 | IVa | − | No |
| 28 | SO-SAU-t304_238 | GCA_018983515.1 | Norway | 6 | t304 | IVa | − | No |
| 29 | RIVM_M043548 | GCA_027733755.1 | Netherlands | 6 | t304 | IVa | − | No |
| 30 | RIVM_M047468 | GCA_042942965.1 | Netherlands | 6 | t304 | IVa | − | No |
| 31 | RIVM_M085903 | GCA_027635885.1 | Netherlands | 6 | t304 | IVa | − | Yes |
| 32 | RIVM_M047301 | GCA_042875335.1 | Netherlands | 6 | t304 | IVa | − | No |
| 33 | RIVM_M047242 | GCA_042873475.1 | Netherlands | 6 | t304 | IVa | − | No |
| 34 | RIVM_M082137 | GCA_027726175.1 | Netherlands | 6 | t304 | IVa | − | No |
| 35 | RIVM_M037546 | GCA_027870655.1 | Netherlands | 6 | t304 | IVa | − | No |
| 36 | RIVM_M084457 | GCA_027646905.1 | Netherlands | 6 | t304 | IVa | − | Yes |
| 37 | RIVM_M086755 | GCA_027773705.1 | Netherlands | 6 | t304 | IVa | − | No |
| 38 | SO-SAU-t304_120 | GCA_018984395.1 | Norway | 6 | t304 | IVa | − | No |
| 39 | SO-SAU-t304_202 | GCA_018983815.1 | Norway | 6 | t304 | IVa | − | No |
| 40 | SO-SAU-t304_273 | GCA_018983285.1 | Norway | 6 | t304 | IVa | − | No |
| 41 | SO-SAU-t304_304 | GCA_018983145.1 | Norway | 6 | t304 | IVa | − | Yes |
| 42 | RIVM_M046603 | GCA_042933075.1 | Netherlands | 6 | t304 | IVa | − | No |
| 43 | SO-SAU-t304_268 | GCA_018983365.1 | Norway | 6 | t304 | IVa | − | No |
| 44 | RIVM_M085014 | GCA_027650805.1 | Netherlands | 6 | t304 | IVa | − | No |
| 45 | RIVM_M088484 | GCA_027756125.1 | Netherlands | 6 | t304 | IVa | − | No |
| 46 | RIVM_M047970 | GCA_027725875.1 | Netherlands | 6 | t304 | IVa | − | Yes |
| 47 | SO-SAU-t304_205 | GCA_018983795.1 | Norway | 6 | t304 | IVa | − | No |
| 48 | RIVM_M087171 | GCA_027755505.1 | Netherlands | 6 | t304 | IVa | − | No |
| 49 | RIVM_M047033 | GCA_042936035.1 | Netherlands | 6 | t304 | IVa | − | No |
| 50 | SO-SAU-t304_164 | GCA_018984015.1 | Norway | 6 | t304 | IVa | − | No |
| 51 | SO-SAU-t304_126 | GCA_018984355.1 | Norway | 6 | t304 | IVa | − | Yes |

## **Supplementary Table S3. Comparative metadata and pairwise core genome SNP distances of the 51 ST6-*Staphylococcus aureus* genomes included in the phylogenomic analysis**

A total of 51 ST6-*Staphylococcus aureus* genomes, including the Egyptian MRSA21-2025 isolate and 50 publicly available reference genomes, were included in the comparative phylogenomic framework. Genomes are ranked according to increasing pairwise core genome SNP distance relative to the study isolate (MRSA21-2025 / SRR37923555). Core SNP distances were calculated from the concatenated Panaroo core genome alignment using Snippy-derived SNP matrices.

The study isolate (MRSA21-2025) was excluded from ranking calculations because pairwise distance to itself equals zero. SNP distance groups were manually assigned for comparative visualization and interpretative purposes.

| Rank by SNP distance | Strain name | Assembly accession | Country of origin | spa type | SCCmec subtype | Core SNP distance to MRSA21-2025 | SNP distance group |
| --- | --- | --- | --- | --- | --- | --- | --- |
| 1 | RIVM_M043548 | GCA_027733755.1 | Netherlands | t304 | IVa | 188 | Closest cluster |
| 2 | SO-SAU-t304_238 | GCA_018983515.1 | Norway | t304 | IVa | 188 | Closest cluster |
| 3 | RIVM_M047970 | GCA_027725875.1 | Netherlands | t304 | IVa | 194 | Closest cluster |
| 4 | SO-SAU-t304_164 | GCA_018984015.1 | Norway | t304 | IVa | 195 | Closest cluster |
| 5 | RIVM_M091081 | GCA_027771485.1 | Netherlands | t304 | IVa | 195 | Closest cluster |
| 6 | SO-SAU-t304_120 | GCA_018984395.1 | Norway | t304 | IVa | 199 | Closest cluster |
| 7 | RIVM_M085903 | GCA_027635885.1 | Netherlands | t304 | IVa | 201 | Closely related |
| 8 | RIVM_M084818 | GCA_027632845.1 | Netherlands | t304 | IVa | 203 | Closely related |
| 9 | RIVM_M047766 | GCA_042932485.1 | Netherlands | t304 | IVa | 205 | Closely related |
| 10 | RIVM_M085205 | GCA_027630665.1 | Netherlands | t304 | IVa | 206 | Closely related |
| 11 | RIVM_M085200 | GCA_027648325.1 | Netherlands | t304 | IVa | 208 | Closely related |
| 12 | RIVM_M089074 | GCA_027759845.1 | Netherlands | t304 | IVa | 210 | Closely related |
| 13 | RIVM_M084457 | GCA_027646905.1 | Netherlands | t304 | IVa | 212 | Closely related |
| 14 | SO-SAU-t304_304 | GCA_018983145.1 | Norway | t304 | IVa | 214 | Closely related |
| 15 | RIVM_M038599 | GCA_027706345.1 | Netherlands | t304 | IVa | 217 | Intermediate cluster |
| 16 | SO-SAU-t304_279 | GCA_018983245.1 | Norway | t304 | IVa | 219 | Intermediate cluster |
| 17 | RIVM_M089421 | GCA_027772605.1 | Netherlands | t304 | IVa | 221 | Intermediate cluster |
| 18 | RIVM_M047900 | GCA_027718925.1 | Netherlands | t304 | IVa | 223 | Intermediate cluster |
| 19 | RIVM_M047531 | GCA_042885885.1 | Netherlands | t304 | IVa | 224 | Intermediate cluster |
| 20 | RIVM_M086737 | GCA_027768905.1 | Netherlands | t304 | IVa | 226 | Intermediate cluster |
| 21 | RIVM_M048996 | GCA_027720465.1 | Netherlands | t304 | IVa | 228 | Intermediate cluster |
| 22 | RIVM_M048555 | GCA_027642105.1 | Netherlands | t304 | IVa | 229 | Intermediate cluster |
| 23 | SO-SAU-t304_202 | GCA_018983815.1 | Norway | t304 | IVa | 232 | Intermediate cluster |
| 24 | SO-SAU-t304_273 | GCA_018983285.1 | Norway | t304 | IVa | 234 | Intermediate cluster |
| 25 | RIVM_M046603 | GCA_042933075.1 | Netherlands | t304 | IVa | 236 | Intermediate cluster |
| 26 | SO-SAU-t304_268 | GCA_018983365.1 | Norway | t304 | IVa | 239 | Intermediate cluster |
| 27 | RIVM_M085014 | GCA_027650805.1 | Netherlands | t304 | IVa | 241 | Intermediate cluster |
| 28 | RIVM_M088484 | GCA_027756125.1 | Netherlands | t304 | IVa | 243 | Intermediate cluster |
| 29 | SO-SAU-t304_205 | GCA_018983795.1 | Norway | t304 | IVa | 246 | Intermediate cluster |
| 30 | RIVM_M087171 | GCA_027755505.1 | Netherlands | t304 | IVa | 249 | Intermediate cluster |
| 31 | RIVM_M047033 | GCA_042936035.1 | Netherlands | t304 | IVa | 251 | Intermediate cluster |
| 32 | SO-SAU-t304_126 | GCA_018984355.1 | Norway | t304 | IVa | 254 | Intermediate cluster |
| 33 | RIVM_M047767 | GCA_042932505.1 | Netherlands | t304 | IVa | 257 | Intermediate cluster |
| 34 | RIVM_M047468 | GCA_042942965.1 | Netherlands | t304 | IVa | 260 | Intermediate cluster |
| 35 | RIVM_M047301 | GCA_042875335.1 | Netherlands | t304 | IVa | 263 | Intermediate cluster |
| 36 | RIVM_M047242 | GCA_042873475.1 | Netherlands | t304 | IVa | 266 | Intermediate cluster |
| 37 | RIVM_M082137 | GCA_027726175.1 | Netherlands | t304 | IVa | 269 | Intermediate cluster |
| 38 | RIVM_M037546 | GCA_027870655.1 | Netherlands | t304 | IVa | 272 | Intermediate cluster |
| 39 | RIVM_M086755 | GCA_027773705.1 | Netherlands | t304 | IVa | 276 | Divergent cluster |
| 40 | RIVM_M041780 | GCA_027824535.1 | Netherlands | t304 | IVa | 280 | Divergent cluster |
| 41 | SO-SAU-t304_153 | GCA_018984155.1 | Norway | t304 | IVa | 284 | Divergent cluster |
| 42 | RIVM_M088076 | GCA_027759005.1 | Netherlands | t304 | IVa | 289 | Divergent cluster |
| 43 | RIVM_M047103 | GCA_042904085.1 | Netherlands | t304 | IVa | 294 | Divergent cluster |
| 44 | RIVM_M085206 | GCA_027648385.1 | Netherlands | t304 | IVa | 299 | Divergent cluster |
| 45 | RIVM_M086260 | GCA_027652965.1 | Netherlands | t4403 | IVa | 305 | Divergent cluster |
| 46 | RIVM_M084446 | GCA_027632305.1 | Netherlands | t304 | IVa | 311 | Divergent cluster |
| 47 | ST6 (CC5) — Korea A | GCA_054559635.1 | South Korea | t304 | IVa | 324 | Highly divergent |
| 48 | ST6 (CC5) — Korea B | GCA_054559695.1 | South Korea | t304 | IVa | 329 | Highly divergent |
| 49 | SO-SAU-t304_251 | GCA_018983475.1 | Norway | t304 | IVa | 348 | Highly divergent |
| 50 | RIVM_M042172 | GCA_027818655.1 | Netherlands | t304 | IVa | 367 | Most divergent |

## **Supplementary Table S4. Pairwise core genome SNP distance statistics across the comparative ST6 dataset**

| Comparison group | Number of pairwise comparisons | Minimum SNPs | Maximum SNPs | Mean ± SD | Median |
| --- | --- | --- | --- | --- | --- |
| MRSA21-2025 vs reference genomes | 50 | 188 | 367 | 224.7 ± 36.9 | 217 |
| Among 50 reference genomes only | 1,225 | 0 | 300 | 148.2 ± 43.9 | 143 |
| Complete dataset (51 genomes) | 1,275 | 0 | 367 | 151.2 ± 46.1 | 145 |

Pairwise core genome SNP distances were calculated from concatenated core genome alignments generated using Snippy and Panaroo. The MRSA21-2025 isolate demonstrated moderate genomic divergence relative to publicly available ST6 reference genomes while remaining within the broader ST6 lineage-associated phylogenetic structure.
