# Supplementary material for: Comparative phylogenomic and long-read genomic characterization of an Egyptian ST6-MRSA-IVa clinical isolate within a globally conserved multidrug-resistant lineage
Source: Front Microbiol. 2026 Jun 8;17:1855574. doi: 10.3389/fmicb.2026.1855574 (PMC13284069; doi:10.3389/fmicb.2026.1855574)
Supplement: Supplementary file 2 [file Data_Sheet_1.PDF]

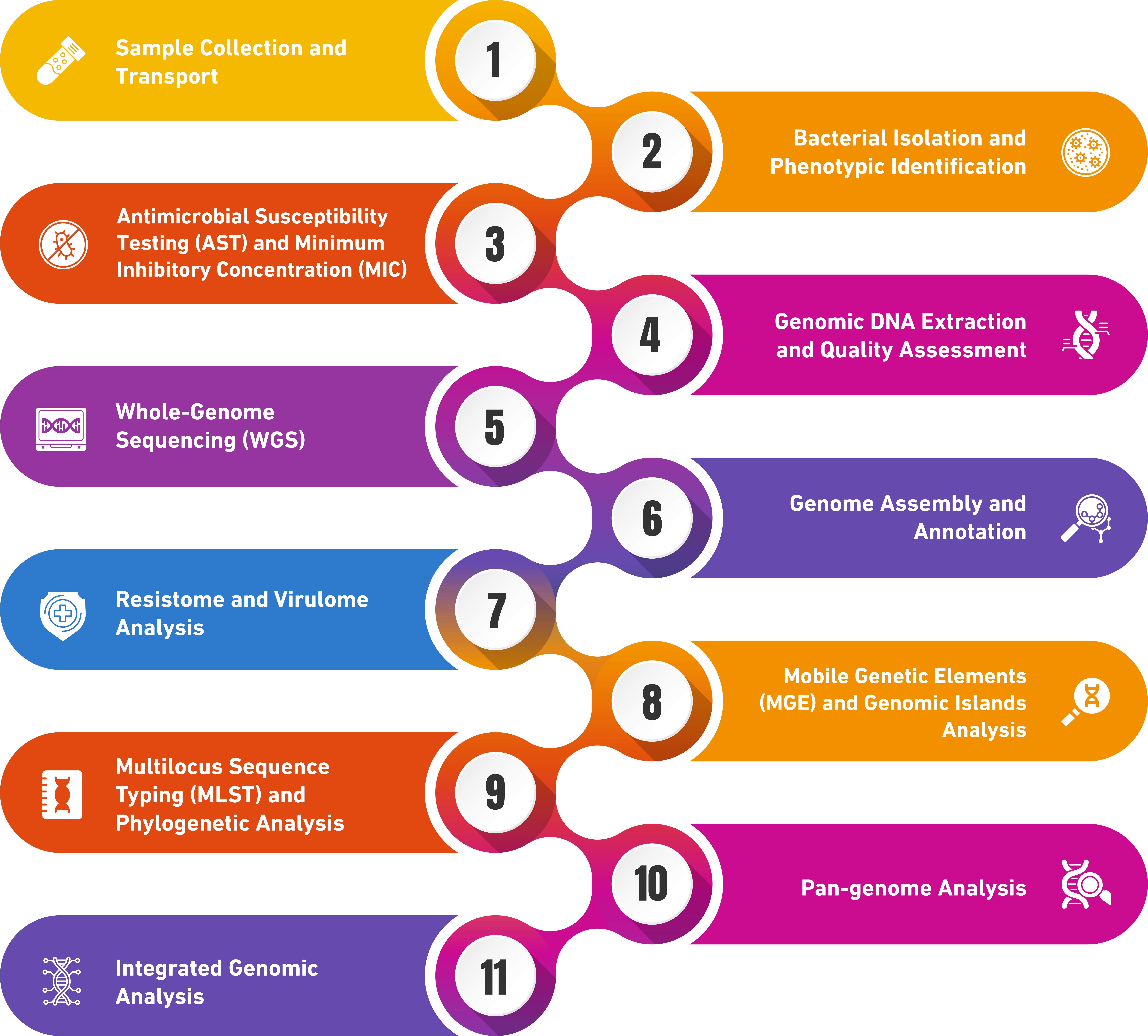
1
Sample Collection and Transport
2
Bacterial Isolation and Phenotypic Identification
3
Antimicrobial Susceptibility Testing (AST) and Minimum Inhibitory Concentration (MIC)
4
Genomic DNA Extraction and Quality Assessment
5
Whole-Genome Sequencing (WGS)
6
Genome Assembly and Annotation
7
Resistome and Virulome Analysis
8
Mobile Genetic Elements (MGE) and Genomic Islands Analysis
9
Multilocus Sequence Typing (MLST) and Phylogenetic Analysis
10
Pan-genome Analysis
11
Integrated Genomic Analysis
