# Supplementary figures and images for: Comparative phylogenomic and long-read genomic characterization of an Egyptian ST6-MRSA-IVa clinical isolate within a globally conserved multidrug-resistant lineage
Source: Front Microbiol. 2026 Jun 8;17:1855574. doi: 10.3389/fmicb.2026.1855574 (PMC13284069; doi:10.3389/fmicb.2026.1855574)

Figure 2A

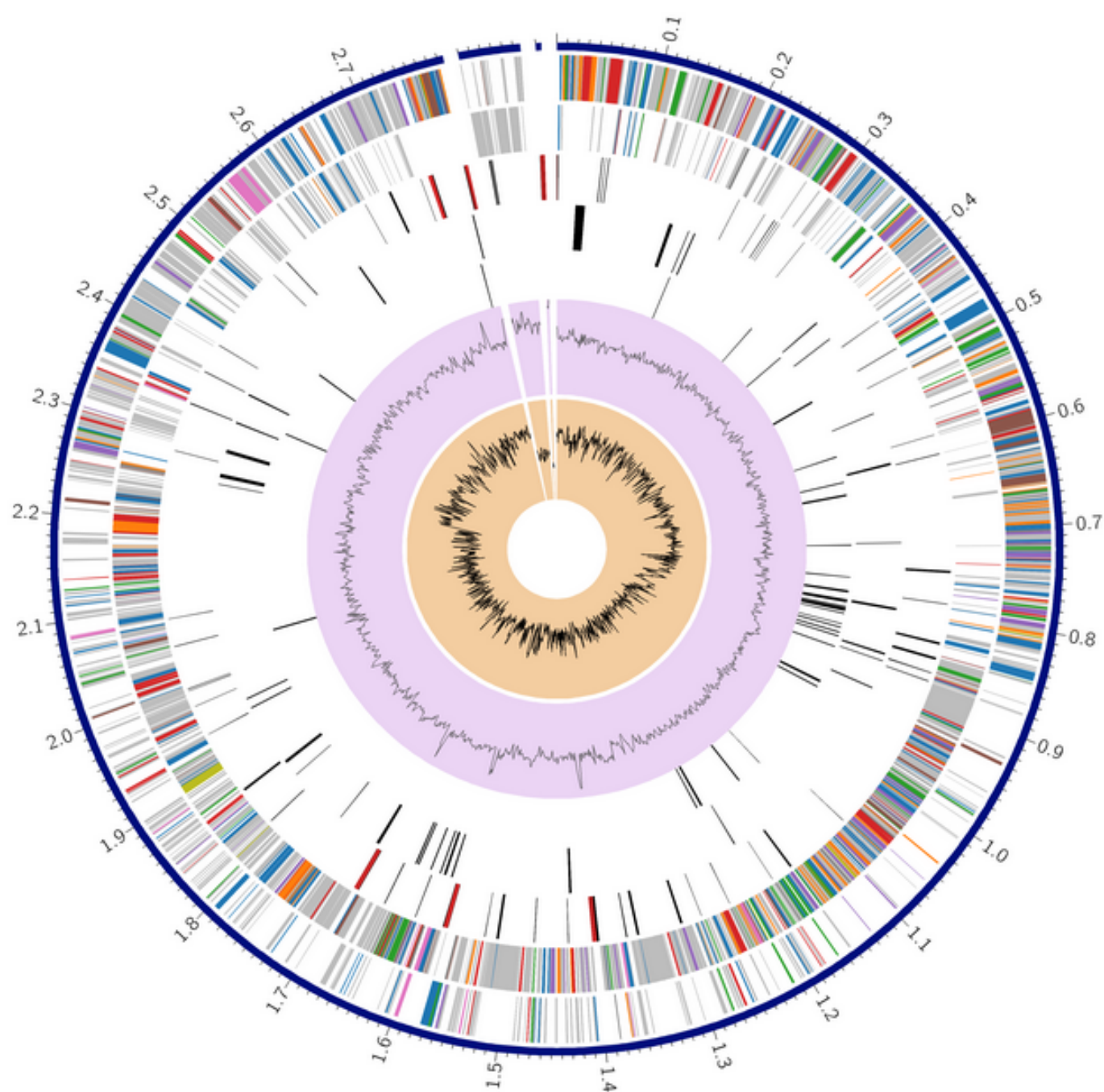

Supplement: Supplementary file 3 [file Data_Sheet_2.PDF]

**Figure 2B**

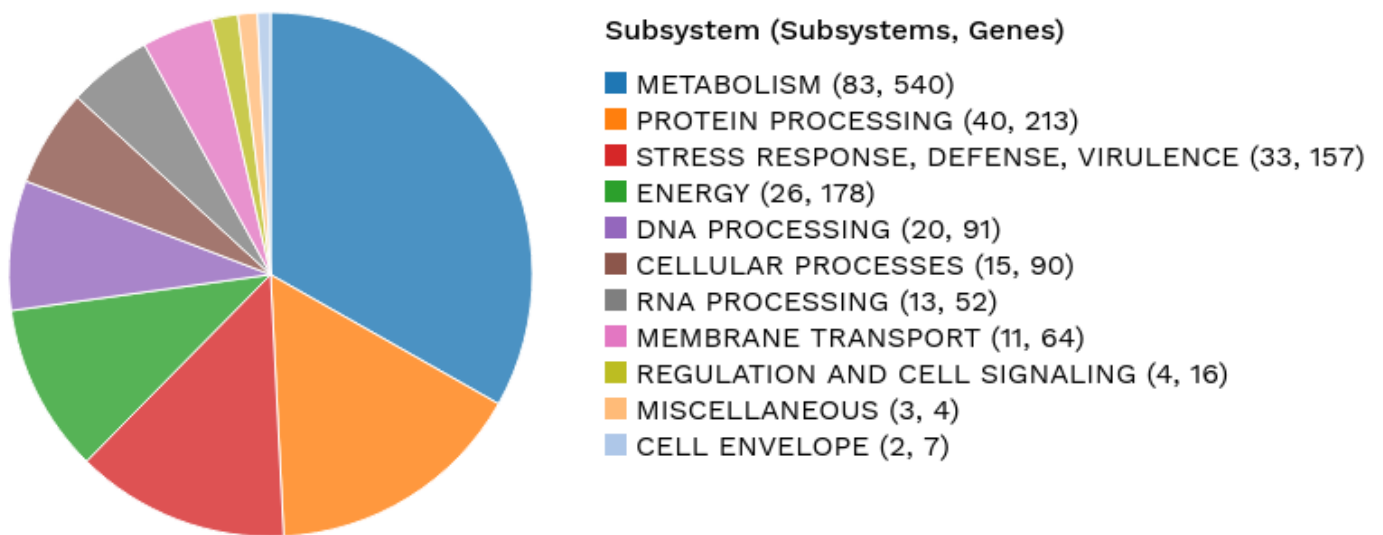

Supplement: Supplementary file 4 [file Data_Sheet_3.PDF]

Figure 4 · AMR · Virulence · IS-family profile across 51 ST6 genomes

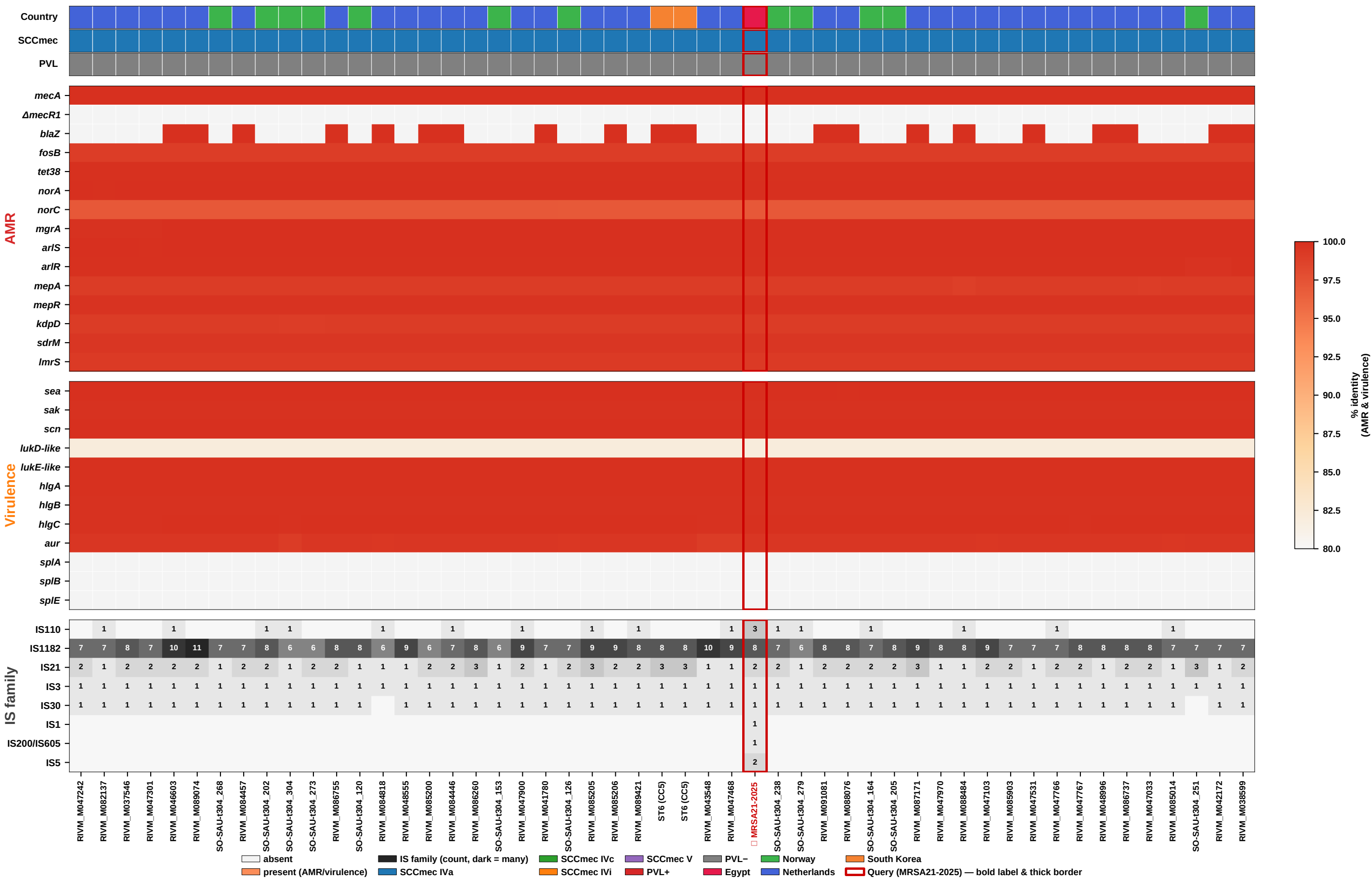

Supplement: Supplementary file 6 [file Data_Sheet_5.PDF]

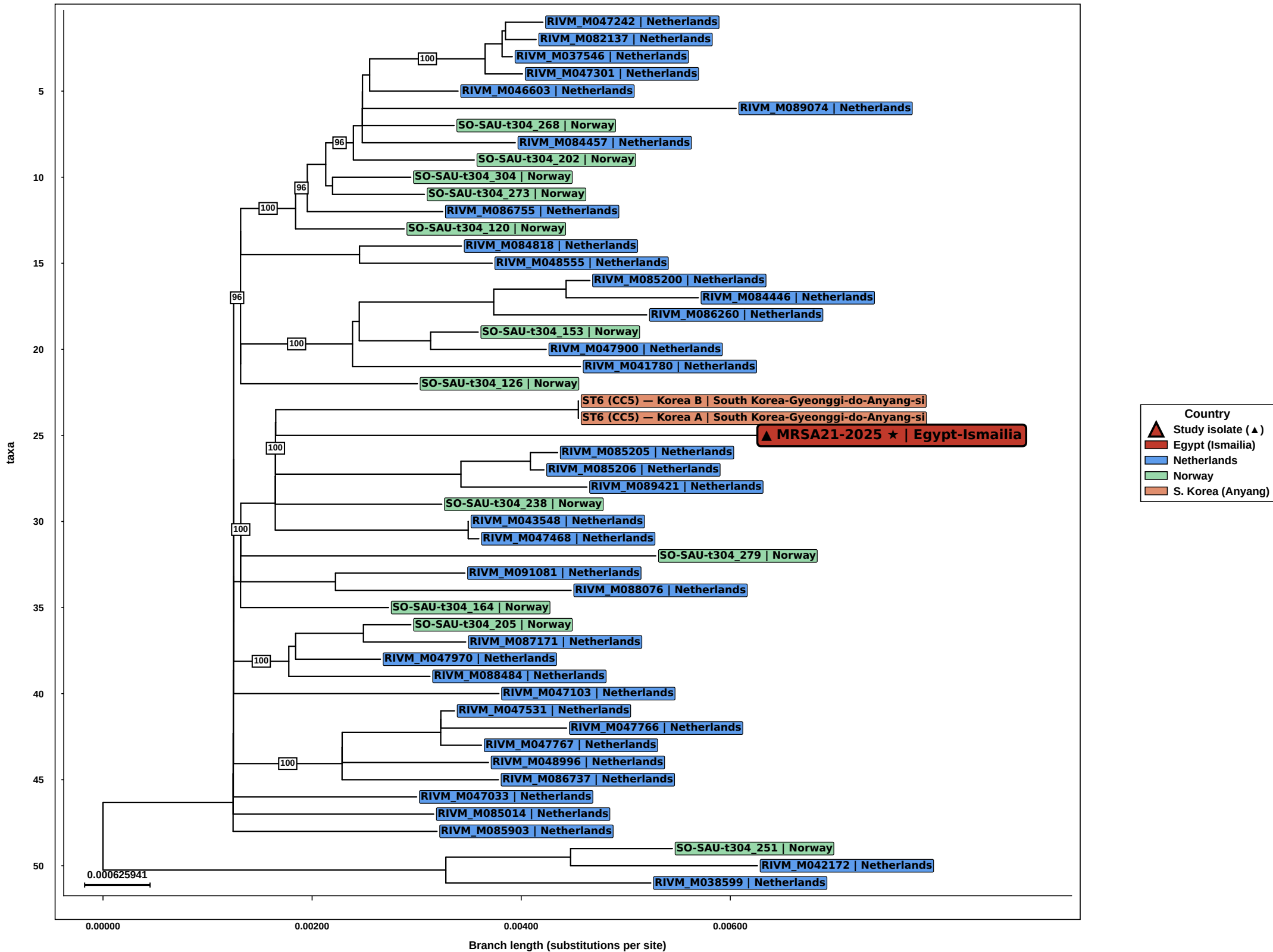

Supplement: Supplementary file 7 [file Data_Sheet_6.PDF]

# Core genome SNP distance relationships among comparative ST6-MRSA genomes

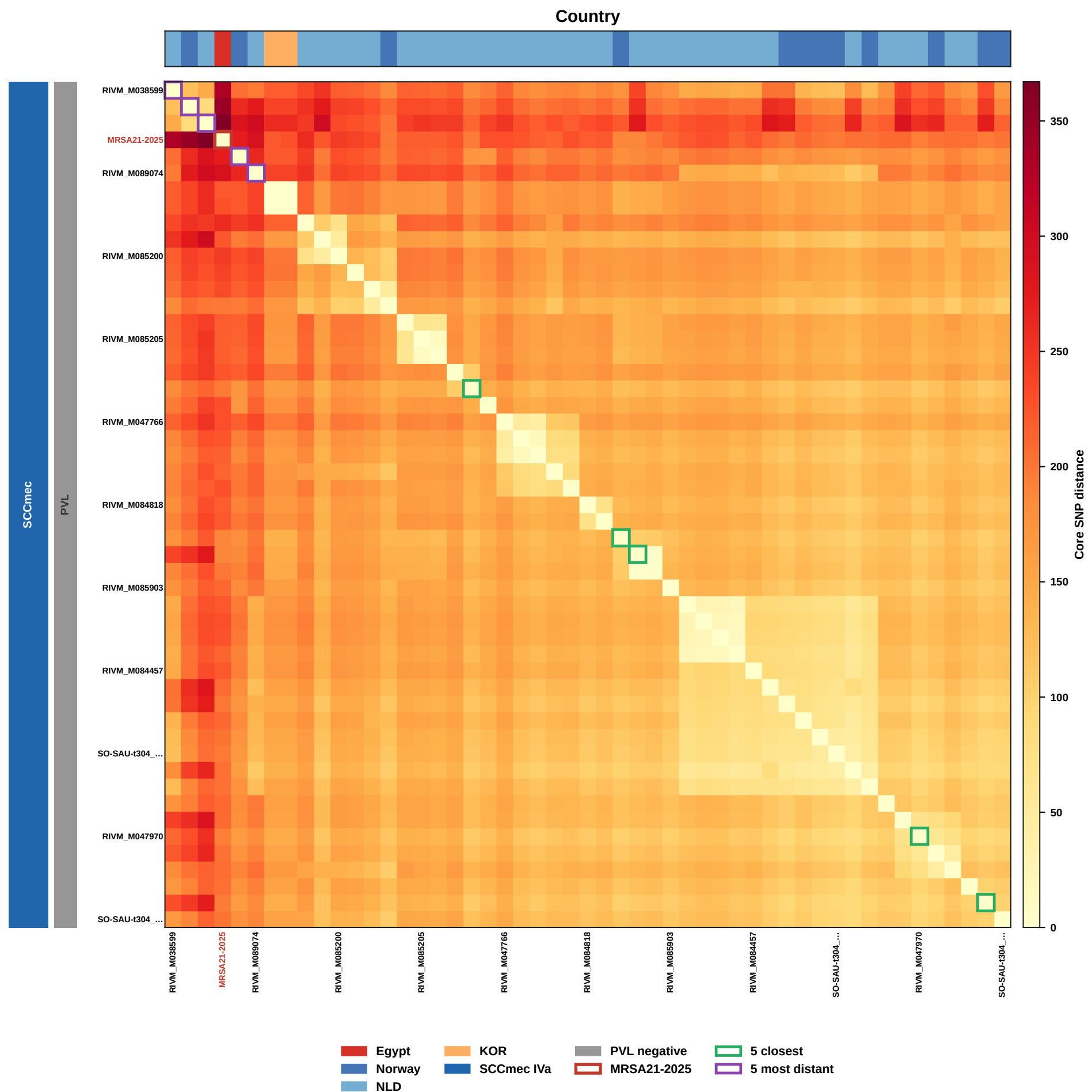

Supplement: Supplementary file 9 [file Data_Sheet_8.PDF]

Supplementary Figure S2

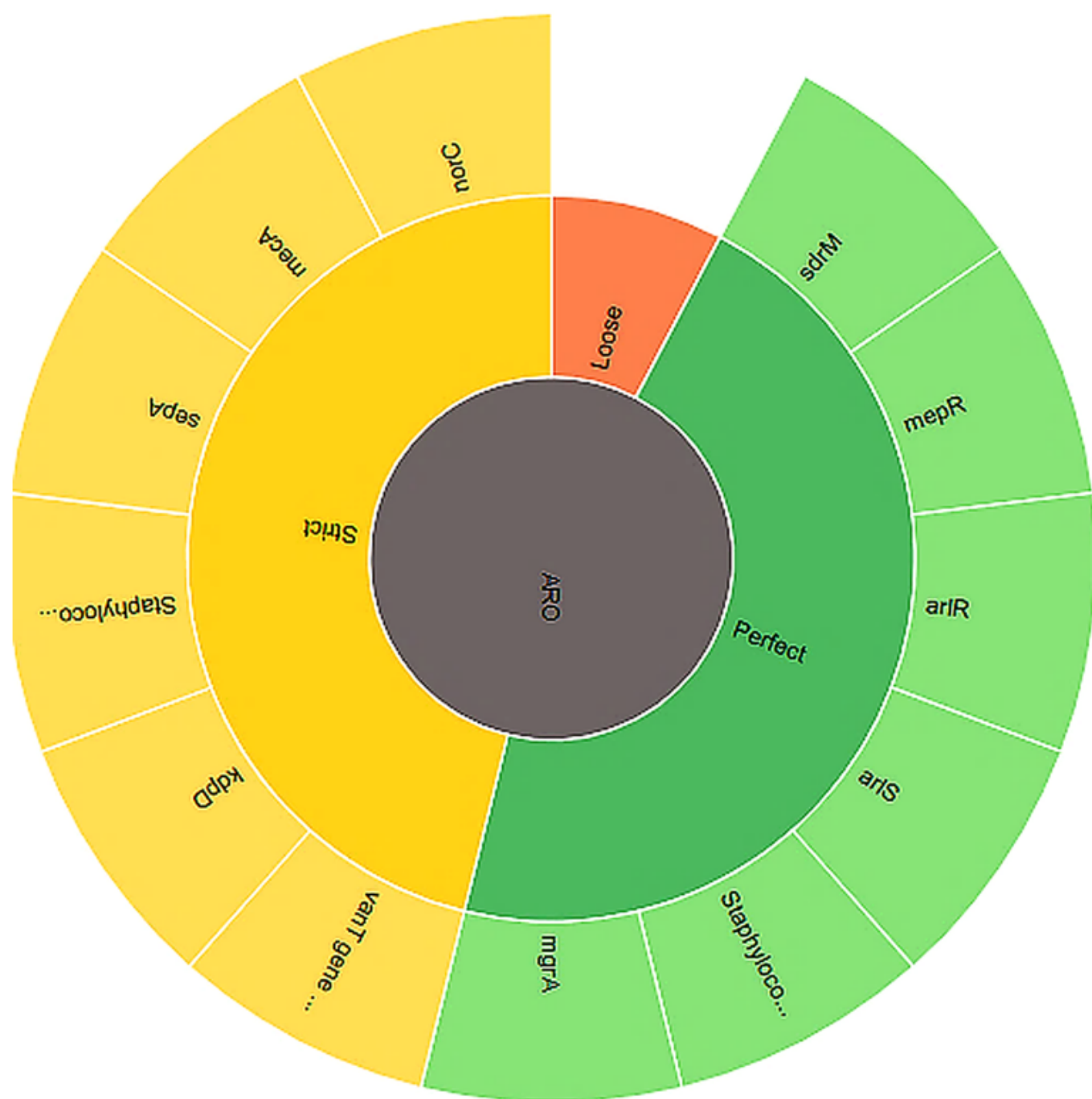

Supplement: Supplementary file 11 [file Data_Sheet_10.PDF]
