# Supplementary material for: Comparative phylogenomic and long-read genomic characterization of an Egyptian ST6-MRSA-IVa clinical isolate within a globally conserved multidrug-resistant lineage
Source: Front Microbiol. 2026 Jun 8;17:1855574. doi: 10.3389/fmicb.2026.1855574 (PMC13284069; doi:10.3389/fmicb.2026.1855574)
Supplement: Supplementary file 5 [file Data_Sheet_4.PDF]

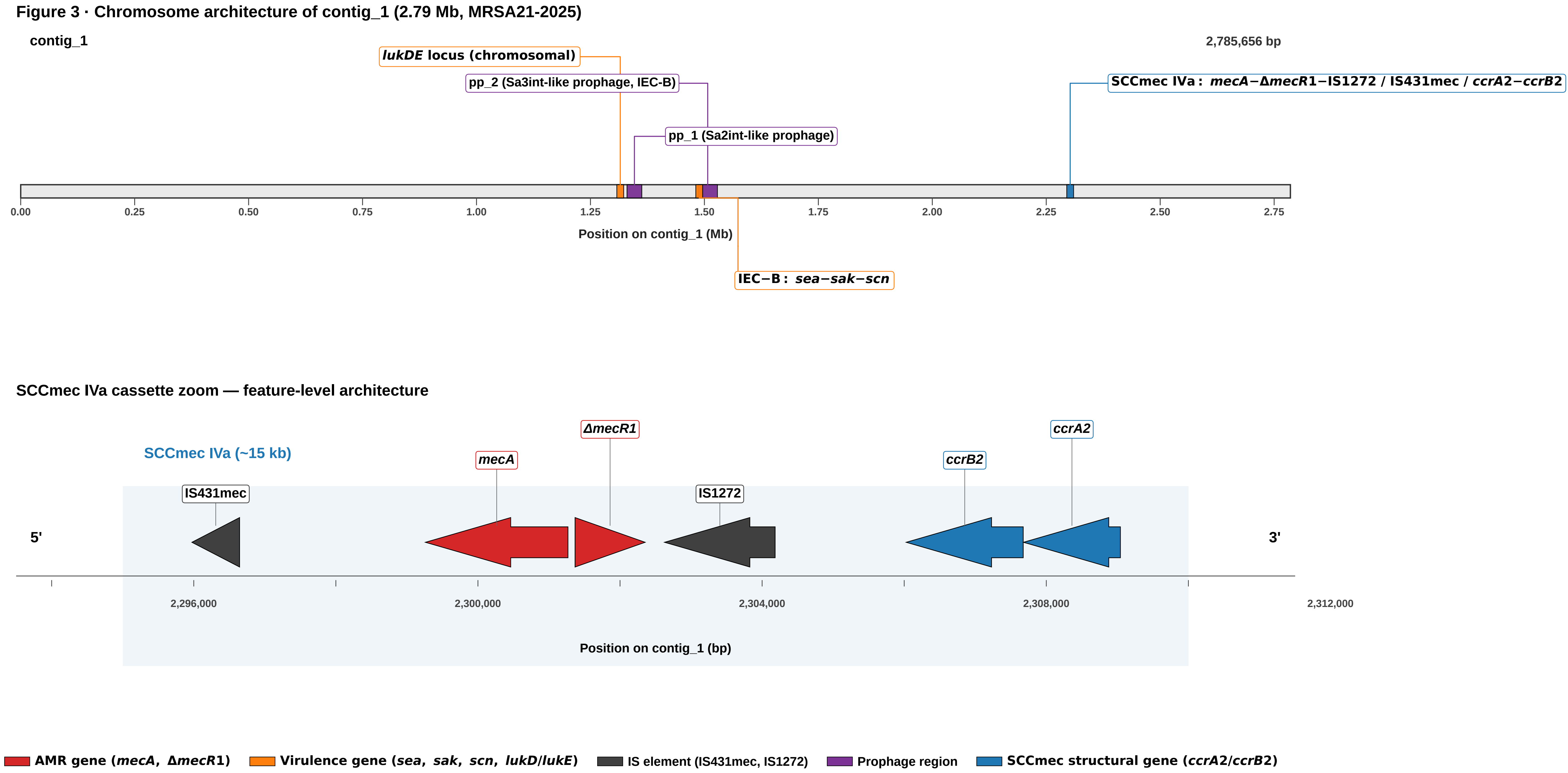
Figure 3 · Chromosome architecture of contig_1 (2.79 Mb, MRSA21-2025)
contig_1
2,785,656 bp
lukDE locus (chromosomal)
pp_2 (Sa3int-like prophage, IEC-B)
SCCmec IVa : mecA−ΔmecR1−IS1272 / IS431mec / ccrA2−ccrB2
pp_1 (Sa2int-like prophage)
0.00
0.25
0.50
0.75
1.00
1.25
1.50
1.75
2.00
2.25
2.50
2.75
Position on contig_1 (Mb)
IEC−B : sea−sak−scn
SCCmec IVa cassette zoom — feature-level architecture
ΔmecR1
SCCmec IVa (~15 kb)
mecA
ccrB2
ccrA2
IS431mec
IS1272
5'
3'
2,296,000
2,300,000
2,304,000
2,308,000
2,312,000
Position on contig_1 (bp)
AMR gene (mecA, ΔmecR1)
Virulence gene (sea, sak, scn, lukD/lukE)
IS element (IS431mec, IS1272)
Prophage region
SCCmec structural gene (ccrA2/ccrB2)
