# Supplementary material for: Comparative phylogenomic and long-read genomic characterization of an Egyptian ST6-MRSA-IVa clinical isolate within a globally conserved multidrug-resistant lineage
Source: Front Microbiol. 2026 Jun 8;17:1855574. doi: 10.3389/fmicb.2026.1855574 (PMC13284069; doi:10.3389/fmicb.2026.1855574)

Whole-genome average nucleotide identity (ANI) relationships among ST6-MRSA genomes

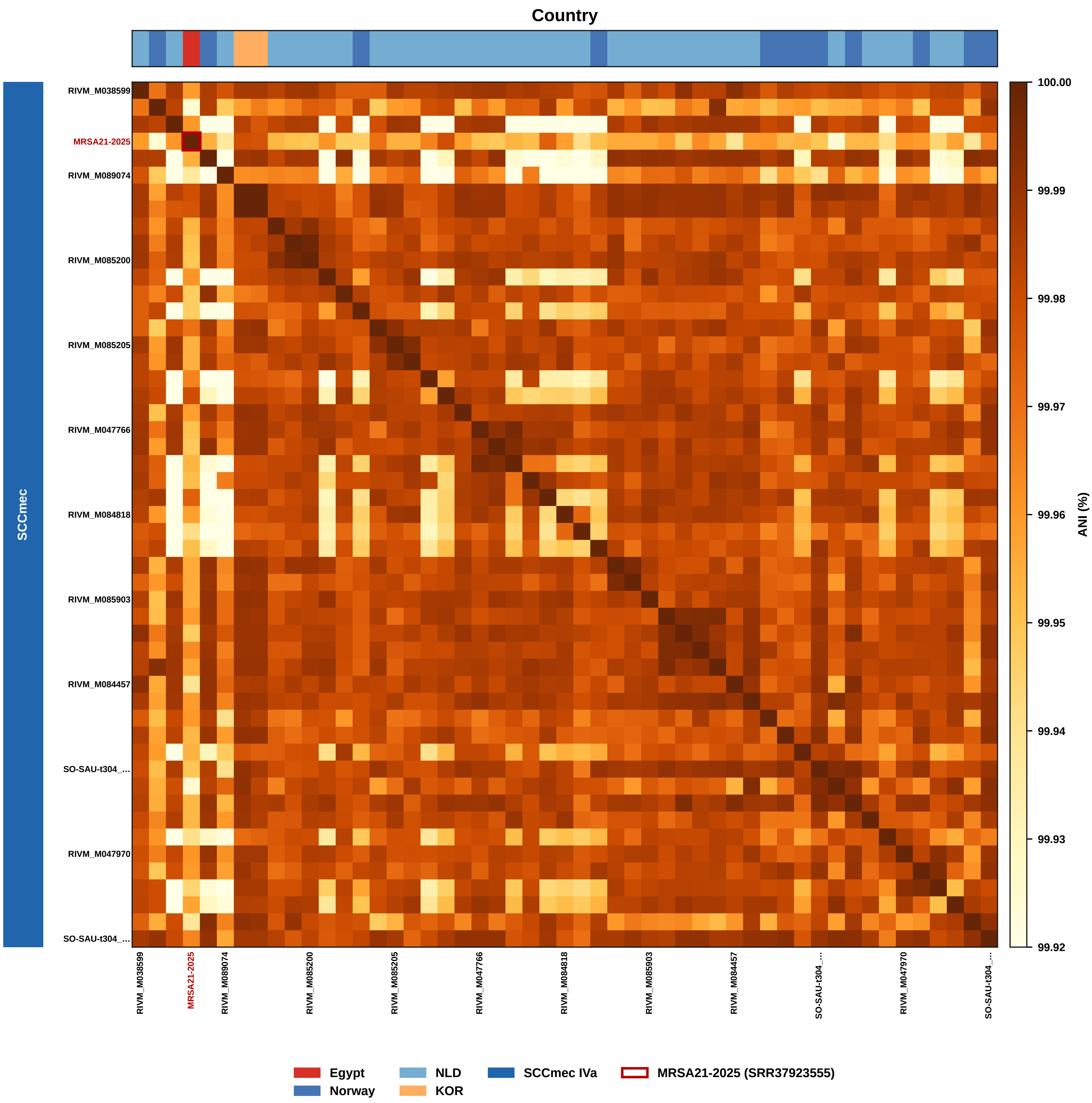

Supplement: Supplementary file 8 [file Data_Sheet_7.PDF]
