# Supplementary material for: Comparative phylogenomic and long-read genomic characterization of an Egyptian ST6-MRSA-IVa clinical isolate within a globally conserved multidrug-resistant lineage
Source: Front Microbiol. 2026 Jun 8;17:1855574. doi: 10.3389/fmicb.2026.1855574 (PMC13284069; doi:10.3389/fmicb.2026.1855574)
Supplement: Supplementary file 10 [file Data_Sheet_9.PDF]

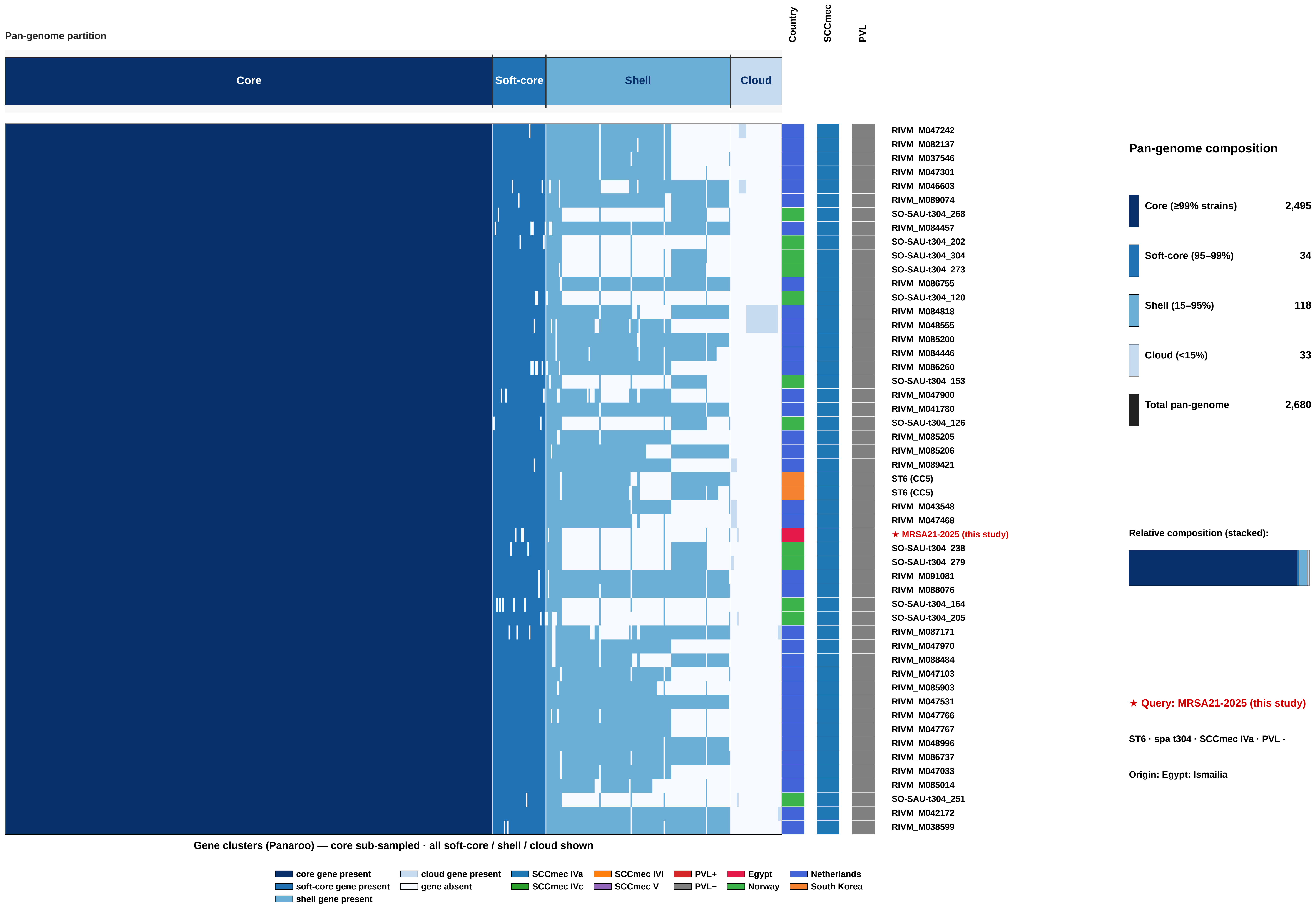

Pan-genome partition
Core
Soft-core
Shell
Cloud
Country
SCCmec
PVL
RIVM_M047242
RIVM_M082137
RIVM_M037546
RIVM_M047301
RIVM_M046603
RIVM_M089074
SO-SAU-t304_268
RIVM_M084457
SO-SAU-t304_202
SO-SAU-t304_304
SO-SAU-t304_273
RIVM_M086755
SO-SAU-t304_120
RIVM_M084818
RIVM_M048555
RIVM_M085200
RIVM_M084446
RIVM_M086260
SO-SAU-t304_153
RIVM_M047900
RIVM_M041780
SO-SAU-t304_126
RIVM_M085205
RIVM_M085206
RIVM_M089421
ST6 (CC5)
ST6 (CC5)
RIVM_M043548
RIVM_M047468
★ MRSA21-2025 (this study)
SO-SAU-t304_238
SO-SAU-t304_279
RIVM_M091081
RIVM_M088076
SO-SAU-t304_164
SO-SAU-t304_205
RIVM_M087171
RIVM_M047970
RIVM_M088484
RIVM_M047103
RIVM_M085903
RIVM_M047531
RIVM_M047766
RIVM_M047767
RIVM_M048996
RIVM_M086737
RIVM_M047033
RIVM_M085014
SO-SAU-t304_251
RIVM_M042172
RIVM_M038599
Pan-genome composition
Core (≥99% strains) 2,495
Soft-core (95–99%) 34
Shell (15–95%) 118
Cloud (<15%) 33
Total pan-genome 2,680
Relative composition (stacked):
★ Query: MRSA21-2025 (this study)
ST6 · spa t304 · SCCmec IVa · PVL -
Origin: Egypt: Ismailia
Gene clusters (Panaroo) — core sub-sampled · all soft-core / shell / cloud shown
core gene present
soft-core gene present
shell gene present
cloud gene present
gene absent
SCCmec IVa
SCCmec IVc
SCCmec IVi
SCCmec V
PVL+
PVL−
Egypt
Norway
Netherlands
South Korea
